# Immune responses to O-specific polysaccharide (OSP) in North American adults infected with *Vibrio cholerae* O1 Inaba

**DOI:** 10.1371/journal.pntd.0007874

**Published:** 2019-11-19

**Authors:** Motaher Hossain, Kamrul Islam, Meagan Kelly, Leslie M. Mayo Smith, Richelle C. Charles, Ana A. Weil, Taufiqur Rahman Bhuiyan, Pavol Kováč, Peng Xu, Stephen B. Calderwood, Jakub K. Simon, Wilbur H. Chen, Michael Lock, Caroline E. Lyon, Beth D. Kirkpatrick, Mitchell Cohen, Myron M. Levine, Marc Gurwith, Daniel T. Leung, Andrew S. Azman, Jason B. Harris, Firdausi Qadri, Edward T. Ryan

**Affiliations:** 1 Division of Infectious Diseases, Massachusetts General Hospital, Boston, Massachusetts, United States of America; 2 Infectious Diseases Division, International Centre for Diarrhoeal Disease Research, Bangladesh (icddr,b), Dhaka, Bangladesh; 3 Harvard Medical School, Boston, Massachusetts, United States of America; 4 National Institute of Diabetes, Digestive and Kidney Diseases (NIDDK), Laboratory of Bioorganic Chemistry (LBC), National Institutes of Health, Bethesda, Maryland, United States of America; 5 Merck & Co., Inc., Kenilworth, New Jersey, United States of America; 6 Center for Vaccine Development, University of Maryland School of Medicine, Baltimore, Maryland, United States of America; 7 PaxVax, Inc., Redwood City, California, United States of America; 8 Vaccine Testing Center, Departments of Medicine and Microbiology and Molecular Genetics, University of Vermont College of Medicine, Burlington, Vermont, United States of America; 9 Cincinnati Children’s Hospital Medical Center, and the Department of Pediatrics, University of Cincinnati College of Medicine, Cincinnati, Ohio, United States of America; 10 Division of Infectious Diseases, University of Utah School of Medicine, Salt Lake City, Utah, United States of America; 11 Department of Epidemiology, Johns Hopkins Bloomberg School of Public Health, Baltimore, Maryland, United States of America; 12 Department of Pediatrics, Harvard Medical School, Boston, Massachusetts, United States of America; 13 Department of Immunology and Infectious Disease, Harvard T.H. Chan School of Public Health, Boston, Massachusetts, United States of America; Institut Pasteur, FRANCE

## Abstract

**Background:**

Antibodies targeting O-specific polysaccharide (OSP) of *Vibrio cholerae* may protect against cholera; however, little is known about this immune response in infected immunologically naïve humans.

**Methodology:**

We measured serum anti-OSP antibodies in adult North American volunteers experimentally infected with *V*. *cholerae* O1 Inaba El Tor N16961. We also measured vibriocidal and anti-cholera toxin B subunit (CtxB) antibodies and compared responses to those in matched cholera patients in Dhaka, Bangladesh, an area endemic for cholera.

**Principal findings:**

We found prominent anti-OSP antibody responses following initial cholera infection: these responses were largely IgM and IgA, and highest to infecting serotype with significant cross-serotype reactivity. The anti-OSP responses peaked 10 days after infection and remained elevated over baseline for ≥ 6 months, correlated with vibriocidal responses, and may have been blunted in blood group O individuals (IgA anti-OSP). We found significant differences in immune responses between naïve and endemic zone cohorts, presumably reflecting previous exposure in the latter.

**Conclusions:**

Our results define immune responses to O-specific polysaccharide in immunologically naive humans with cholera, find that they are largely IgM and IgA, may be blunted in blood group O individuals, and differ in a number of significant ways from responses in previously humans. These differences may explain in part varying degrees of protective efficacy afforded by cholera vaccination between these two populations.

**Trial registration number:**

ClinicalTrials.gov NCT01895855.

## Author summary

Cholera is an acute, secretory diarrheal disease caused by *Vibrio cholerae* O1. There is a growing body of evidence that immune responses targetting the O-specific polysaccharide (OSP) of *V*. *cholerae* are associated with protecton against cholera. Despite this, little is known about immune responses targeting OSP in immunologically naive individals. Cholera affects populations in severely resource-limited areas. To address this, we assessed anti-OSP immune responses in North American volunteers experimentally infected with wild type *V*. *cholerae* O1 El Tor Inaba strain N16961. We found that antibody responses were largely IgM and IgA, cross-reacted to both Inaba and Ogawa serotypes, and correlated with vibriocidal responses. We found no association of responses to severity of disease, but did find that blood group O individuals mounted lower IgA fold-changes to OSP than did non-blood group O individuals. Individuals with blood group O are at particular risk for severe cholera, and are less well protected against cholera following oral vaccination. We also compared anti-OSP responses in previously unexposed individuals to responses in matched endemic zone patients, and found a number of significant differences. Such differences may explain in part the varying degrees of protective efficacy afforded by cholera vaccination between these two populations.

## Introduction

Cholera is a diarrheal disease caused by *Vibrio cholerae* serogroups O1 or (rarely) O139 and occurs most commonly among people living in low and middle income countries [1]. *V*. *cholerae* has over 200 serogroups, but only the O1 and O139 serogroups cause epidemic and endemic cholera. The O1 serogroup is further divided into El Tor and classical biotypes and subdivided into two major serotypes, Inaba and Ogawa, on the basis of a number of genotypic and phenotypic characteristics [2]. The Ogawa serotype differs from the Inaba serotype by having a 2-*O*-methyl group in the non-reducing terminal sugar of the O-specific polysaccharide (OSP) portion of the lipopolysaccharide (LPS); the methyl group is not present in the Inaba serotype [3]. The prevalence of Inaba and Ogawa serotypes often fluctuates over time in endemic areas and the serotype can switch between Ogawa and Inaba during an outbreak of cholera [4].

OSP is a component of *V*. *cholerae* LPS [5]. Importantly, protection against cholera is serogroup-specific. *V*. *cholerae* O1 infection confers no cross-protection from *V*. *cholerae* O139 infection and vice versa [6–8]. However, residual membrane proteins remaining in standard LPS preparations complicate the assessment of immune responses to specific serotypes [9]. To address this, we have purified *V*. *cholerae* O1 OSP [5] and previously assessed immune responses in cholera patients and vaccinees residing in endemic zones such as Bangladesh and Haiti [9–17]. We found that anti-OSP and anti-LPS responses strongly correlate, and that OSP adsorption of cholera convalescent serum eliminates the vibriocidal response, a marker associated with protection against cholera [9–20]. Similar to vibriocidal antibodies, baseline serum antibody levels targeting OSP are associated with protection against cholera in household contacts of cholera index patients in Bangladesh [12]. We have also recently shown that development of immune responses targeting OSP following oral cholera vaccination is associated with protection against diarrhea following subsequent experimental infection [14].

As such, there is a growing body of evidence that immune responses targeting OSP are involved in mediating protection against cholera; however, little is known about this immune response in immunologically naïve humans. To assess anti-OSP responses in such individuals, we measured serum anti-OSP immune responses in adult North American volunteers experimentally infected with *Vibrio cholerae* O1. We also assessed vibriocidal and immune responses targeting the B subunit (CtxB) of cholera toxin (CT). Interestingly, immune responses that target CT and CtxB have not been highly correlated with protection against cholera [21–23]. We also compared anti-OSP, CtxB and vibriocidal responses in experimentally infected North American volunteers to those induced in age, gender, blood group, and serotype-matched *V*. *cholerae* O1-infected cholera patients in Dhaka, Bangladesh, an area endemic for cholera. Immunogenicity and protective efficacy of oral cholera vaccines have varied when evaluated in cholera endemic zone versus naïve populations, and we hypothesized that a direct comparison of responses in these cohorts might identify possibly relevant differences.

## Methods and materials

### Ethics statement

This study used anonymized samples from a previously reported clinical trial (http://clinicaltrials.gov/show/NCT01895855) [24] and samples collected at the International Centre for Diarrhoeal Disease Research, Bangladesh (icddr,b), Dhaka, Bangladesh. This study was approved by the Partners-Massachusetts General Hospital Institutional Review Board, Boston, Massachusetts, USA and the Institutional Review Board of icddr,b, Dhaka, Bangladesh. Written informed consent was obtained from all participants [14, 24].

### Enrollment of North American volunteers and sample collection

North American healthy adult volunteers (18–45 years of age) were enrolled in a previously described randomized, double blind, placebo-controlled phase 3 clinical trial [14, 24, 25] (Table 1).

**Table 1:** Characteristics of North American experimentally-infected volunteers and Bangladeshi naturally-infected cholera patients.

Characteristics	North American volunteers (n = 38)	Bangladeshi cholera patients (n = 38)
Number of male (%)	21 (55.3)	21 (55.3)
Median age in year (25^th^, 75^th^ percentile)	30.5 (25.3, 35)	29.5 (25, 36)
Blood group O (%)	18 (47.4)	18 (47.4)

These volunteers were experimentally infected with 1x10^5^ CFU of *V*. *cholerae* O1 El Tor Inaba strain N16961 and blood samples were collected before infection (designated as day 0) and at days 10, 28, and 90 or 170 after infection [14, 24, 25]. The specimens used in our current analysis were from participants that received placebo and were infected with N16961 at 10-days or 3-months after placebo-vaccine ingestion in the parent efficacy trial [24]. Of 38 participants analyzed, day 90 samples were available from the 16 subjects who were infected with *V*. *cholerae* 10-days after placebo ingestion; samples from day 170 were available from the remaining 22 participants who were infected 3 months after placebo ingestion. Each subject was treated with ciprofloxacin (500 mg twice daily for 5 days) if they reached 5.0 L of cumulative diarrheal stool volume or on day 4 post experimental infection, whichever occurred first [24].

### Enrollment of Bangladeshi patients and sample collection

All the 38 Bangladeshi patients included in this study were adults 18 to 45 years of age (Table 1). All 38 were infected with *V*. *cholerae* O1 Inaba and were further selected from the icddr,b hospital by 1:1 matching gender, age (± 2.5 year of the paired mean age) and blood group with the North American volunteers. Patients with severe acute watery diarrhea, stool culture positive for *V*. *cholerae* O1 Inaba, and negative for other pathogens were eligible, as previously described [9, 21]. All cholera patients were treated with intravenous fluid resuscitation and oral antibiotics at the discretion of the attending clinician [9, 21]. Blood samples from these patients were selected to approximate the timing of sample collection for the North American volunteers. Blood samples from Bangladeshi participants included those collected on the second day of hospitalization (day 2) following clinical stabilization and rehydration, and then on days 7, 21 or 30, 90 and 180 in follow up. In the Bangladeshi patient cohort, sample numbers available for this study were n = 38 at day 2 and 7; n = 37 at combined day 21/30, and n = 13 at both day 90 and day 180.

### Immunologic measurements

Stored serum or plasma specimens were used to measure Inaba and Ogawa vibriocidal responses against PIC018 (Inaba) and PIC158 (Ogawa) strains, and IgM, IgA and IgG immune responses against *V*. *cholerae* OSP purified from PIC018 (Inaba) and PIC158 (Ogawa), and CtxB, as previously described [5, 9, 12, 14]. Anti-OSP and anti-CtxB antibody responders were defined as those having a ≥ 2.0-fold increase in ELISA units (normalized kinetic reading) over baseline. Vibriocidal responders were defined as individuals with a ≥ 4-fold increase in reciprocal end-titer over baseline [14].

### Statistical analyses

We estimated the geometric mean at each study time point within each population and accompanying asymptotic 95% confidence intervals for IgM, IgA and IgG anti-OSP and anti-CtxB titers in addition to vibriocidal responses. We compared responses between two time points within each cohort using Wilcoxon signed-rank tests and compared responses between populations using Mann-Whitney U tests. We characterized the linear relationship between OSP responses (log) and vibriocidal titers (log_2_) using Pearson’s correlation coefficient. We used LOESS smoothing, as implemented in the ggplot2 package in R, to visualize trajectories of antibody responses and relationships between responses and diarrhea volume. We used linear regression and generalized additive models to characterize the association between specific antibody responses and diarrhea volume. We used linear regression models to estimate adjusted effect of O-blood group on antibody response. All *P* values were two-tailed, and we used *P* ≤ 0.05 as the threshold for statistical significance. We used GraphPad Prism, version 5.0 (GraphPad Software, Inc., La Jolla, CA) and the R statistical computing language (3.5.2) for all analyses and illustrations.

## Results

### IgM antibody responses to Inaba and Ogawa OSP

**Fig 1:**
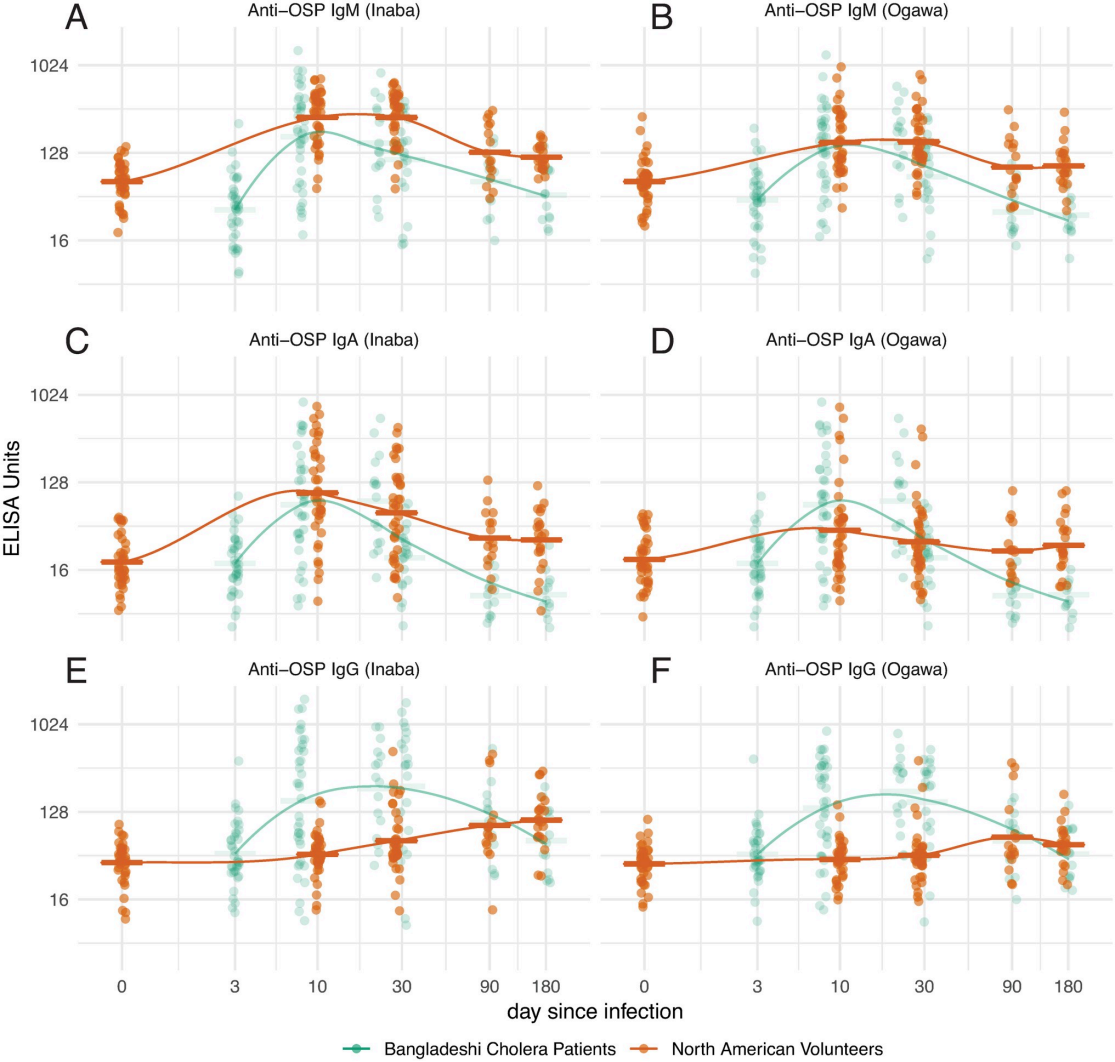
OSP responses over time in North American volunteers (orange) and Bangladeshi cholera patients (green). Dots represent individual responses at each time since infection (jittered) for IgM (A-B), IgA (C-D) and IgG (E-F). Initial data points for Bangladeshi samples assigned day 3 from presumed date of infection. Lines represent LOESS curve fit to response data from each group and horizontal bars represent the geometric mean titer for each time point. Please refer to Supplemental S1–S3 Figs for detailed statistical comparisons among values.

We found significant increases of IgM antibody responses to Inaba OSP at day 10 and the responses remained significantly elevated compared to baseline up to day 170 after experimental infection in North Americans (Fig 1A and S1 Fig; all *P* values for this and all subsequent comparisons are detailed in corresponding Supplemental Figures).

Inaba OSP-specific IgM antibody responses were significantly higher in North Americans than Bangladeshis at days 28, 90 and 180 (Fig 1A; S1 Fig). Anti-Ogawa OSP responses in North Americans mirrored anti-Inaba responses, although of lower magnitude than those targeting Inaba OSP (Fig 1B; S1 Fig). The magnitude of peak anti-Inaba and Ogawa immune responses in Bangladeshis were comparable to one another and had returned to baseline by day 90.

### IgA antibody responses to Inaba and Ogawa OSP

We found significant increases of IgA antibody to Inaba OSP at day 10 compared to baseline and these responses remained significantly elevated up to day 170 in North Americans (Fig 1C; S2 Fig). In Bangladeshis, we found significant IgA immune responses to Inaba OSP at day 7 and these responses remained elevated at the combined day 21/30 time point (Fig 1C; S2 Fig) but decreased to baseline after that. IgA anti-Inaba OSP responses were comparable at day 0, day 7–10 and day 28–30 in North Americans compared to Bangladeshis but were significantly higher in North Americans on day 90 and 170–180 (Fig 1C; S2 Fig). IgA immune responses to Ogawa OSP in North Americans were less prominent than to Inaba, while robust but short-lived IgA anti-Ogawa OSP responses were detected in Bangladeshis, significantly exceeding the response seen in North Americans (Fig 1D; S2 Fig).

### IgG immune responses to Inaba and Ogawa OSP

We found small but significant increases of anti-OSP IgG beginning at day 10 after experimental infection and these responses continued to increase through convalescence in North Americans (Fig 1E; S3 Fig). In Bangladeshis, we found much more significant IgG immune responses to Inaba OSP, including by day 7 after natural infection, and these responses remained significantly elevated up to day 180 (Fig 1E; S3 Fig). IgG immune responses to Ogawa OSP in North Americans were less prominent than to Inaba antigen, while robust IgG anti-Ogawa OSP responses comparable to anti-Inaba IgG responses were detected in Bangladeshis, significantly exceeding the response seen in North Americans (Fig 1F; S3 Fig).

### Antibody responses to CtxB

**Fig 2:**
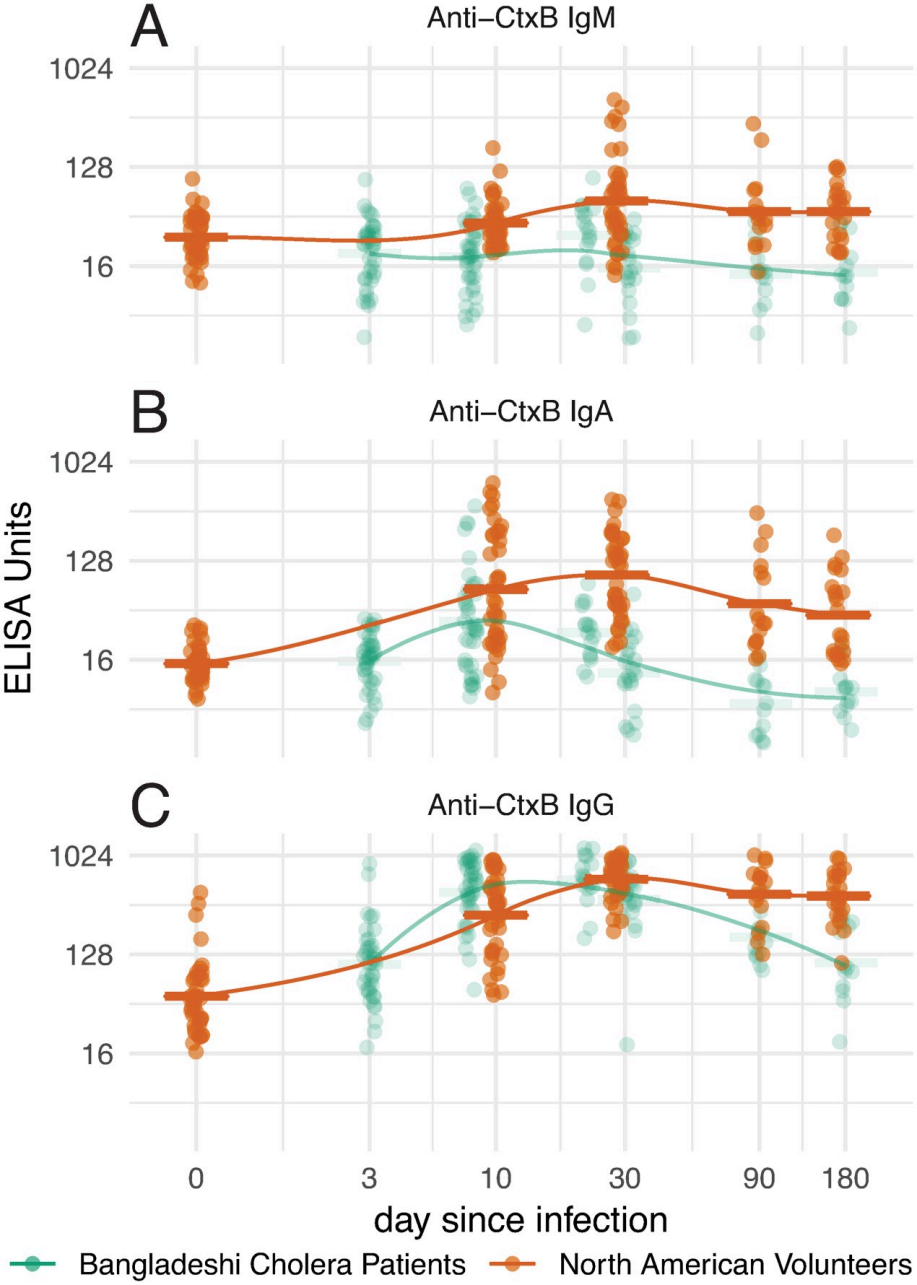
Anti-cholera toxin B (CtxB) responses in North American volunteers (orange) and Bangladeshi cholera patients (green). Dots represent individual responses at each time since infection (jittered) for IgM (A), IgA (B) and IgG (C). Initial data points for Bangladeshi samples assigned day 3 from presumed date of infection. Lines represent LOESS curve fit to response data from each group and horizontal bars represent the geometric mean titer for each time point. Please refer to Supplemental S4 Fig for detailed statistical comparisons among values.

CtxB-specific IgM, IgA, and IgG antibody levels in North Americans were significantly higher at day 10 compared to day 0, and the responses remained significantly elevated up to day 170 (Fig 2A; S4 Fig).

In Bangladeshis, we found IgA and IgG responses to CtxB, but no IgM antibody responses (Fig 2A; S4 Fig). At almost all time points, anti-CtxB IgM, IgA, IgG responses were higher in North Americans than Bangladeshis.

### Vibriocidal responses to *V*. *cholerae* O1 Inaba and Ogawa

**Fig 3:**
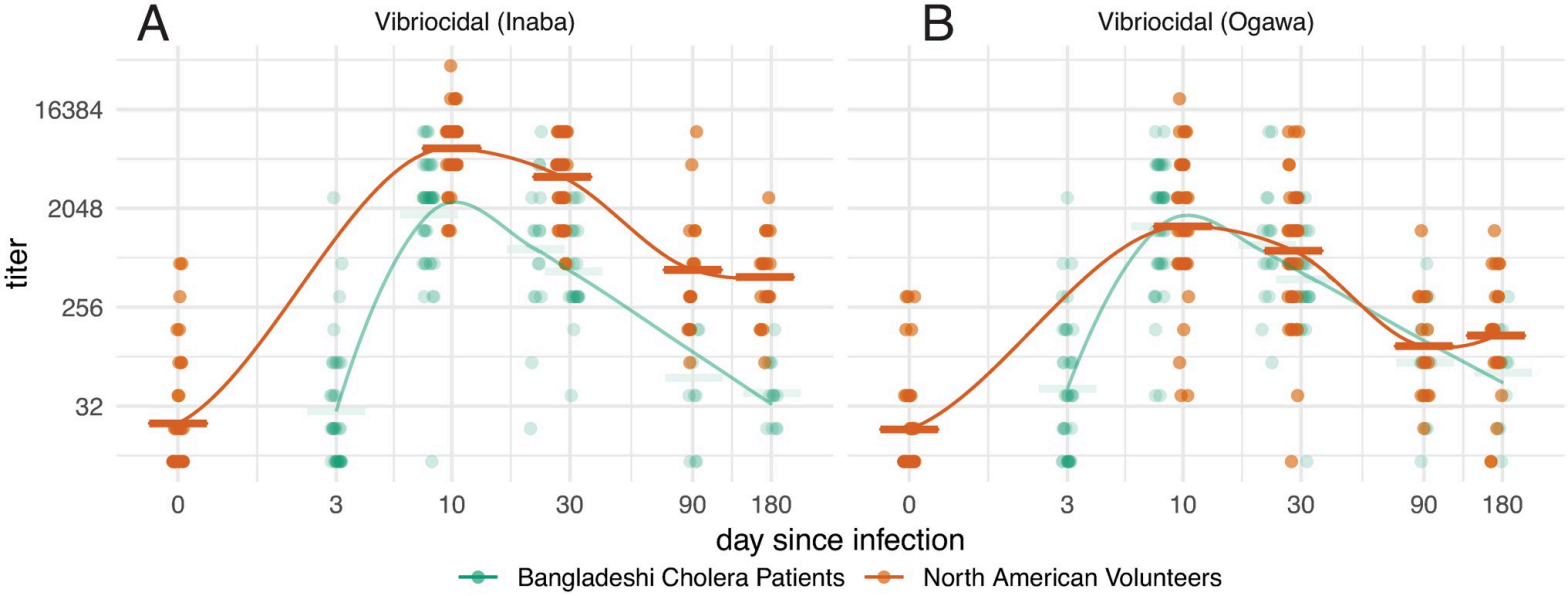
Vibriocidal responses over time in North American volunteers (orange) and Bangladeshi cholera patients (green). Dots represent individual responses at each time since infection (jittered) for vibriocidal titers to Inaba (A) and Ogawa (B) serotypes. Initial data points for Bangladeshi samples assigned day 3 from presumed date of infection. Lines represent LOESS curve fit to response data from each group and horizontal bars represent the geometric mean titer for each time point. Please refer to Supplemental S5 Fig for detailed statistical comparisons among values.

We found a significant increase in the vibriocidal response to *V*. *cholerae* O1 Inaba in North American volunteers that peaked at the first sampled time point after experimental infection (10 days), started to decrease by the next sampled time point (day 28), but remained significantly elevated compared to baseline through the last sampling point (day 170) after experimental infection (Fig 3A; S5 Fig).

In Bangladeshis, the vibriocidal responses to *V*. *cholerae* O1 Inaba also peaked at the first sampled time point (7 days) following infection, started to decrease at the next sampling (day 21), and had returned to baseline by days 90 and 180 (Fig 3A; S5 Fig). The Inaba vibriocidal responses was significantly higher in North Americans than Bangladeshis (Fig 3A; S5A Fig; S1 Table). Both peak and duration of anti-Ogawa vibriocidal responses were lower than Inaba responses in North Americans, while Ogawa and Inaba vibriocidal responses in Bangladeshis were comparable (Fig 3B; S5 Fig; S2 Table). The peak Ogawa vibriocidal response in Bangladeshis was equivalent to that in North Americans (Fig 3B; S5B Fig; S2 Table).

### Correlation of OSP antibody responses with vibriocidal responses

Taking all time points into consideration, Inaba and Ogawa OSP-specific IgM responses most strongly correlated with Inaba and Ogawa vibriocidal responses, respectively, in both North American volunteers and Bangladeshi patients (S6 Fig). OSP-specific IgA responses also correlated strongly, although less than IgM, with vibriocidal responses in both cohorts. There was weak correlation of anti-Inaba but not anti-Ogawa OSP-specific IgG responses and vibriocidal titers in North Americans, but there was significant correlation in Bangladeshis.

### Clinical outcomes

We explored the relationship between baseline, peak and the fold-change of each antibody response (Inaba only for OSP and vibriocidal responses) and total diarrhea volume within the first four days after experimental infection in North American subjects. We identifed no strong relationships through visual inspection (S7–S9 Figs), but noted a potential inverse relationship between peak and fold-change in anti-OSP IgA responses and diarrheal volume; and a positive relationship between peak anti CtxB IgM response and diarrheal volume. Using both linear regression models, including adjustment for O-blood type, we further explored these relationships and estimated no significant relationship between OSP IgA (541 gram reduction in cummulative diarrhea per log increase in peak response over first 10 days, 95% CI -886 to 1969; 300 gram reduction in cummulative diarrhea per fold-change in antibody response over first 10-days, 95% CI -746 to 1347), nor CtxB IgM (716.4 grams of cummulative diarrhea per log increase in peak titer over first 10 days, 95% CI -1012.0 to 2445.0) and diarrheal volume in the first four days after infection. We found similar qualitive results using generalized additive models with splines.

### Effect of blood group

**Fig 4:**
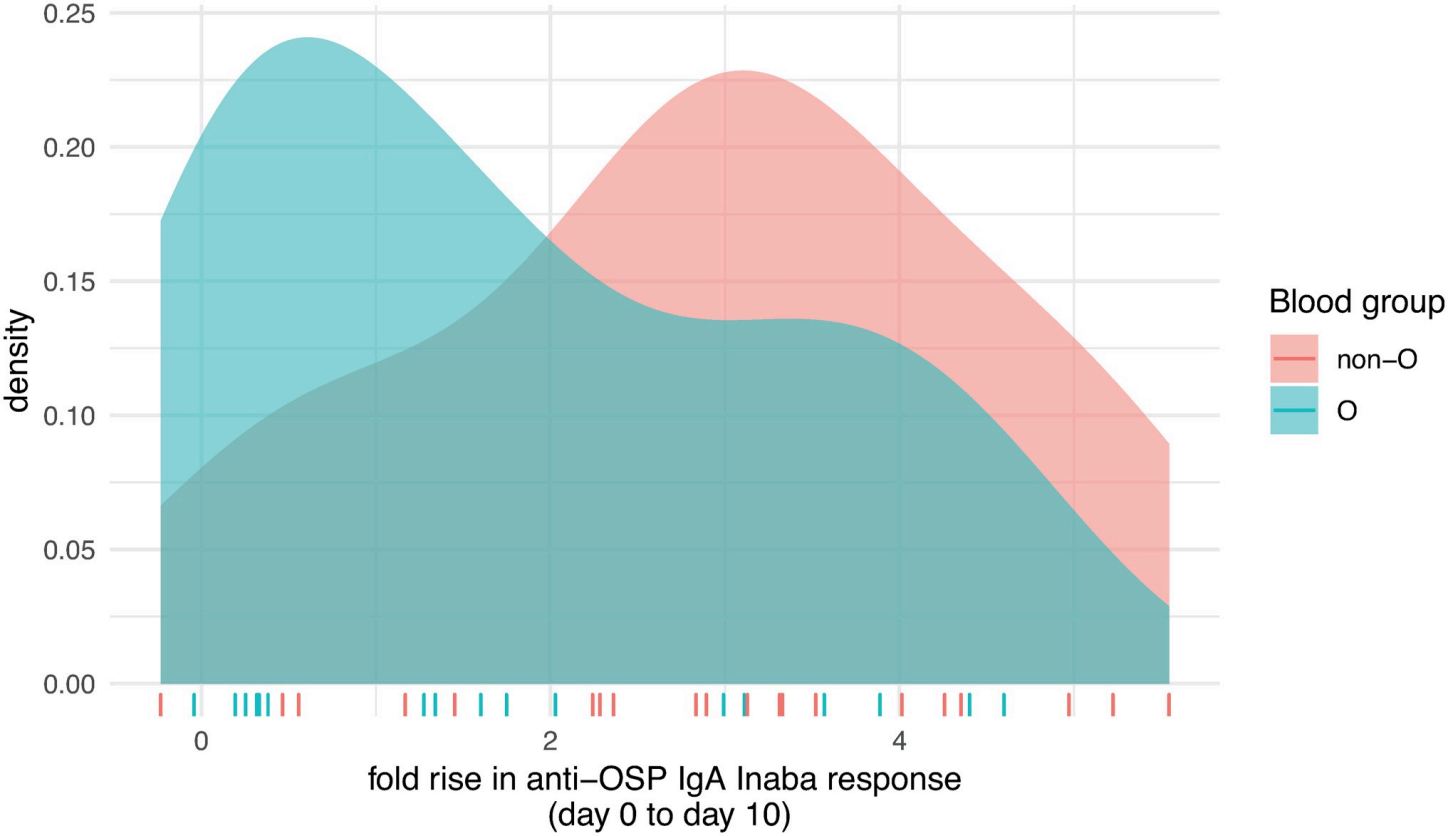
Fold-changes of anti-Inaba OSP IgA responses by O versus non-O blood group status. Distribution of fold-changes of anti-Inaba OSP IgA responses from pre-infection to day 10 following experimental infection of North American volunteers with *V*. *cholerae* O1 by O versus non-O blood group status. Colored ticks on x-axis illustrate the values of the fold-change for each participant.

Although we did not find a difference in mean immune response to OSP by blood group (O versus non-O), we did find that fold-change IgA responses to Inaba OSP were higher in non-O blood group North Americans (median 3.01) than in O group individuals (median 1.47; Fig 4; *P* = 0.048).

We did not see this in the Bangladeshi cohort (S10–S14 Figs). In a linear regression model, we found that non-O blood group North Americans had, on average, 0.92-fold higher fold-change in OSP-IgA response (95% CI -0.66 to 2.06) over the first 10-days compared to O blood group individuals, after controlling for timing of antibiotic use and baseline OSP IgA titer. We found no differences in vibriocidal or CtxB responses by blood group.

## Discussion

In this study, we focused our analysis on anti-OSP, vibriocidal, and anti-CtxB immune responses for a number of reasons. The vibriocidal response has historically been the response most closely associated with protection against cholera, although its association is probably correlative rather than mechanistic [26]. The vibriocidal response assessed *in vitro* is largely comprised of serum IgM targeting OSP [9–11, 13, 14, 27]. We assessed anti-OSP responses since there is a growing body of evidence that immune responses targeting OSP may be mechanistically involved in protection against cholera [12, 14], and we assessed immune responses to CT since it is a critical virulence determinant of cholera that is immunologically processed as a T cell-dependent antigen, while OSP is immunologically processed as a T cell-independent antigen [22].

Anti-OSP and vibriocidal responses often cross-react in Ogawa and Inaba antigenic assays using samples from endemic zone patients [9]. Whether this is due to prior exposure has been uncertain. Here, we detected anti-Ogawa OSP responses in previously unexposed humans infected with Inaba *V*. *cholerae*. This corroborates previous vibriocidal analyses in experimental volunteers [14], but is the first confirmation in previously unexposed individuals of this cross-reactivity to OSP, the antigen that mediates serogroup specificity, with protection against cholera being serogroup specific [6]. The structure of Inaba and Ogawa serotypes is highly similar, comprised of repetitive perosamines [28], only differing by the presence or absence of a methyl group on the terminal saccharide [3].

We found that vibriocidal responses in North American volunteers most highly correlated with IgM and IgA anti-OSP responses, and that these correlations were highest to the homologous serotype to experimental infection (Inaba). In comparison, we found that Bangladeshi patients had higher correlation coefficients than matched North Americans for all antibody isotypes, and that Ogawa correlations were comparable to Inaba correlations, despite the patients being infected with Inaba serotype *V*. *cholerae*. These data might suggest that repetitive exposure to *V*. *cholerae* of Bangladeshis results in cross-serotype responses, as well as antibody isotype and functional maturation.

Antibody responses to CtxB in North American volunteers was largely IgG, with a lower level IgA response, and a minimal IgM response. Although it is unlikely that North American volunteers would have been previously exposed to wild type epidemic *V*. *cholerae*, it is possible that some may have been exposed to enterotoxigenic *E*. *coli* (ETEC) that expresses heat labile toxin (LT) that shares structural similarities and immunologic cross-reactivity with CT [29, 30]. As such, we cannot exclude a boosting anti-LT/CT effect following prior exposure; however, CT is not only a potent enterotoxin, but also a potent immunoadjuvant and immunogen, and the immune responses we detected would be consistent with a T-cell dependent immune response and antibody isotype maturation and class switching.

With regard to severity of disease resulting from experimental infection, we did not find that it correlated with peak, fold-change, or duration of any of the immune responses that we assessed, although there was a trend toward lower fold increases in anti-OSP IgA responses and volume of diarrhea. These observations may suggest that other factors may impact the clinical and immune variability seen during cholera. Such factors might include host genetic or nutritional variability, the microbiota or blood grouping [19, 31–35]. Indeed, blood group O is associated with an increased severity of cholera [36–38]. Interestingly, in our present analysis of blood group impact on immune responses, we found that individuals of blood group O had a lower fold increase in IgA responses to OSP than non-O-group individuals in early convalescence despite having comparable vibriocidal responses. We have previously found that individuals who are Lewis blood type (Le-/-) also have severe diarrhea during cholera and have a blunted LPS IgA response during early convalescence [39]. OSP is the antigen within LPS that gives sero-specific protection against cholera. Why individuals of blood group O and Lewis (-/-) are more likely to develop severe cholera is not fully understood; however, one hypothesis is that there may be an interaction of blood group saccharides of both the ABO group as well as the Lewis group and binding of cholera toxin [40, 41]. Blood group antigens have also been associated with variations on innate immune responses during intestinal infections [40]. Interestingly, individuals of blood group O have previously been reported to have lower protection compared to non-O recipients following killed oral cholera vaccination despite comparable vibriocidal responses [42, 43]. Our current results may in part explain this apparent discrepancy, since IgA may not meaningfully contribute to functional vibriocidal activity observed *in vitro* (IgA does not bind complement through the classical system) [44]. We therefore hypothesize that both ABO and Lewis blood group antigens may be involved in modifying mucosal IgA immune responses against *V*. *cholerae* OSP/LPS, perhaps through the impact of initial innate immune handling, and that these lower mucosal IgA responses lead to the observed lower protective efficacy following killed OCV in blood group O individuals compared to non-O individuals.

In this study we also had a unique opportunity to compare immune responses between North American immunologically naïve volunteers and matched Bangladeshi adults residing in a cholera endemic urban setting. Although we found that Bangladeshi patients naturally infected with *V*. *cholerae* O1 Inaba also developed prominent anti-OSP, vibriocidal, and anti-CtxB antibody responses, we detected important differences in these responses between these two cohorts. Interestingly, the magnitude of the vibriocidal responses, the percent responder frequency, and the duration of elevation were all higher in immunologically naïve North Americans experimentally infected with wild type *V*. *cholerae* than matched Bangladeshi controls. This is despite the fact that baseline vibriocidal values were comparable between the two groups. One possible explanation is that Bangladeshi adults had pre-existing mucosal immunity not reflected by baseline plasma vibriocidal assessment in this highly cholera endemic area, and that this pre-existing mucosal immunity blunted immunological processing and subsequent serum responses [20, 45]. Alternatively, chronic mucosal inflammation that is common in low-resource settings (environmental enteropathy) or differing microbiota could have altered responses [32, 46], or the oral inoculum of Bangladeshi patients could have been lower than the 10^5^ CFU administered to North American volunteers. Our findings of blunted vibriocidal responses in endemic zone residents mirror those seen when attenuated live oral cholera vaccine strains have been administered in low resource setting [47, 48].

We found that although anti-OSP IgM and IgA responses occurred in Bangladeshi adults, the magnitude and duration of these responses were higher in North American volunteers, mirroring the vibriocidal responses. We also found that Bangladeshi adults recovering from cholera developed much more prominent IgG responses to OSP than did North American volunteers. Indeed, in Bangladeshi adults, the vibriocidal response highly correlated with IgG as well as IgM and IgA anti-OSP responses to both Inaba and Ogawa serotypes, while in North Americans, it only weakly correlated to IgG targeting Inaba, but not Ogawa OSP. Presumably this reflects previous exposure in Bangladeshis. We also found that Bangladeshis developed anti-Ogawa OSP and vibriocidal responses that were very comparable to anti-Inaba responses, and these anti-Ogawa responses were much more prominent than those detected in North Americans. These differences would also all be consistent with previous exposure to *V*. *cholerae* Inaba and Ogawa in the Bangladeshi study participants.

Our study has a number of limitations. We focused on immune responses to *V*. *cholerae* O1 in adults, and immune responses in children may well be quite different. Although children in a endemic zone may be immunologically naïve, they also respond less prominently to T cell-independent antigens, especially young children. Second, we did not include direct assessment of mucosal immune responses. Third, North American volunteers were experimentally infected with a fixed number of bacteria (1x10^5^ CFU of *V*. *cholerae* O1 El Tor Inaba strain N16961), while the infectious dose of naturally infected patients in Bangladesh may differ from patient to patient and circulating strains may vary [49]. Fourth, we largely focused on analysis of immune responses to two important antigens, OSP and CtxB, but did not measure immune responses to other potentially important antigens. Fifth, our study did not include an assessment of memory responses, long-lived plasma cell, or T cell responses. Finally, the time points of samples for North American volunteers and Bangladeshi patients were not exactly the same since the samples were originally collected in unrelated studies and the true times of infection for the Bangladeshi cohort is unknown. Despite these limitations, our data suggest that prominent anti-OSP responses develop following an initial *V*. *cholerae* infection, that cross-serotype antibody responses develop, that anti-OSP IgM and IgA responses most highly correlate with vibriocidal responses following initial exposure, and that blood group O correlates with lower anti-OSP IgA responses. We also found a number of prominent differences in immune responses following cholera in immunological naïve North Americans versus in individuals residing in a highly endemic cholera region in Bangladesh. Whether such differences might impact protection following oral cholera vaccination is currently uncertain.

## Supporting information

S1 Table Inaba vibriocidal GMTs by time point in North American volunteers and Bangladeshi patients. (DOCX)

S2 Table Ogawa vibriocidal GMTs by time point in North American volunteers and Bangladeshi patients. (DOCX)

S1 Fig IgM antibody responses to Inaba and Ogawa OSP in blood of North American volunteers and Bangladeshi patients. In the two cohorts, the numbers of patients with sample measurements are shown below the x axes. (1A) Inaba OSP-specific IgM antibody responses and (1B) Ogawa OSP-specific IgM antibody responses. X axis indicates the time points of samples, and the Y-axis denotes IgM antibody responses. Each single dot indicates an individual antibody response, horizontal bars indicate the geometric mean (GM), and error bars indicate 95% confidence intervals. *P* values represent statistical differences of the mean between the country groups. Asterisks represent statistically significant differences of immune responses within the country group compared to baseline (*** *P ≤* 0.001, ** *P ≤* 0.01, * *P* ≤ 0.05.). Responder frequencies (defined in text) are shown in parentheses below the X-axes.
(TIF)

S2 Fig IgA antibody responses to Inaba and Ogawa OSP in North American volunteers and Bangladeshi patients. In the two cohorts, the numbers of patients with sample measurements are shown below the x axes. (2A) Inaba OSP-specific IgA antibody responses and (2B) Ogawa OSP-specific IgA antibody responses. X axes indicate the time points of samples, while the Y-axes denote IgA antibody responses. Each single dot indicates an individual antibody response, horizontal bars indicate the geometric mean (GM), and error bars indicate 95% confidence intervals. *P* values represent statistical differences of the mean between the country groups. Asterisks represent statistically significant differences of immune responses compared to baseline within the country group (*** *P ≤* 0.001, ** *P ≤* 0.01, * *P* ≤ 0.05). Responder frequencies (defined in text) are shown in parentheses below the X-axes.
(TIF)

S3 Fig IgG antibody responses to Inaba and Ogawa OSP in North American volunteers and Bangladeshi patients. In the two cohorts, the numbers of patients with sample measurements are shown below the x axes. (3A) Inaba OSP-specific IgG antibody responses and (3B) Ogawa OSP-specific IgG antibody responses. X axes indicate the time points of samples, while the Y-axes denote IgG antibody responses. Each single dot indicates an individual antibody response, horizontal bars indicate the geometric mean (GM), and error bars indicate 95% confidence intervals. *P* values represent statistical differences of the mean between the country groups. Asterisks represent statistically significant differences of immune responses compared to baseline within country group (*** *P ≤* 0.001, ** *P ≤* 0.01, * *P* ≤ 0.05). Responder frequencies (defined in text) are shown in parentheses below the X-axes.
(TIF)

S4 Fig Antibody (IgM, IgA and IgG) responses to CtxB in North American volunteers and Bangladeshi patients. In the two cohorts, the numbers of patients with sample measurements are shown below the x axes. (4A) CtxB-specific IgM antibody responses, (4B) CtxB-specific IgA antibody responses and (4C) CtxB-specific IgG antibody responses. X axes indicate the time points of samples, while the Y-axes denote antibody responses. Each single dot indicates an individual antibody response, horizontal bars indicate the geometric mean (GM), and error bars indicate 95% confidence intervals. *P* values represent statistical differences of the mean between the country groups. Asterisks represent statistically significant differences of immune responses compared to baseline within country group (*** *P ≤* 0.001, ** *P ≤* 0.01, * *P* ≤ 0.05). Responder frequencies (defined in text) are shown in parentheses below the X-axes.
(TIF)

S5 Fig Comparison of vibriocidal antibody responses to *V*. *cholerae* O1 Inaba and Ogawa in North Americans volunteers and Bangladeshi patients. The time points of samples are plotted on the X axis while vibriocidal antibody responses are plotted on the Y-axis. (5A) Inaba vibriocidal antibody responses and (5B) Ogawa vibriocidal antibody responses. Each single dot indicates an individual vibriocidal antibody reciprocal end-dilution titer. Horizontal bars indicate the Geometric Mean (GM) and error bars indicate 95% Confidence Intervals (CI). *P* values represent statistical differences of the mean vibriocidal responses between the two country groups. Asterisks represent statistically significant differences of vibriocidal responses compared to the baseline time point within a country group (*** *P* ≤ 0.001, * *P* ≤ 0.05). Responder frequencies (defined in text) are also presented in parentheses below the X-axes.
(TIF)

S6 Fig Correlation between OSP-responses and vibriocidal responses in North American volunteers (orange) and Bangladeshi cholera patients (green). Lines represent simple linear regression lines with asymptotic 95% confidence intervals shown as grey envelopes. Pearson’s correlation coefficient and corresponding p-values are shown in each panel. Left panels compare Inaba OSP-specific IgM, IgA, and IgG to Inaba vibriocidal responses; Right panels compare Ogawa OSP-specific IgM, IgA, and IgG to Ogawa vibriocidal responses.
(TIF)

S7 Fig Relationship between peak immune response and diarrhea volume in the first four days after experimental infection. Blue lines show smoothed LOESS estimates and 95% confidence intervals.
(TIF)

S8 Fig Relationship between baseline immune value and diarrhea volume in the first four days after experimental infection. Blue lines show smoothed LOESS estimates and 95% confidence intervals.
(TIF)

S9 Fig Relationship between fold-change of immune response and diarrhea volume in the first four days after experimental infection. Blue lines show smoothed LOESS estimates and 95% confidence intervals.
(TIF)

S10 Fig Inaba OSP specific immune responses in blood group O and non-O of North American volunteers’ and Bangladeshi patients. X axis indicates the time points of samples, while Y-axis denotes OSP-specific antibody responses. Each single dot indicates an individual OSP antibody value, horizontal bars indicate the geometric mean (GM), and error bars indicate 95% confidence intervals.
(TIF)

S11 Fig Ogawa OSP specific immune responses in blood group O and non-O of North American volunteers’ and Bangladeshi patients. X axis indicates the time points of samples, while Y-axis denotes OSP-specific antibody responses. Each single dot indicates an individual OSP antibody value, horizontal bars indicate the geometric mean (GM), and error bars indicate 95% confidence intervals.
(TIF)

S12 Fig CtxB Specific immune responses in blood group O and non-O of North American volunteers’ and Bangladeshi patients. X axis indicates the time points of samples, while Y-axis denotes CtxB-specific antibody responses. Each single dot indicates an individual CtxB antibody value, horizontal bars indicate the geometric mean (GM), and error bars indicate 95% confidence intervals.
(TIF)

S13 Fig Comparison of Inaba vibriocidal responses between blood group O and non-O in North Americans volunteers and Bangladeshi patients. X axis indicates the time points of samples, while Y-axis denotes Inaba vibriocidal antibody responses. Each single dot indicates an individual vibriocidal antibody titer, horizontal bars indicate the geometric mean (GM), and error bars indicate 95% confidence intervals.
(TIF)

S14 Fig Comparison of Ogawa vibriocidal responses between blood group O and non-O in North Americans volunteers and Bangladeshi patients. X axis indicates the time points of samples, while Y-axis denotes Ogawa vibriocidal antibody responses. Each single dot indicates an individual vibriocidal antibody titer, horizontal bars indicate the geometric mean (GM), and error bars indicate 95% confidence intervals.
(TIF)
