# Supplementary material for: Immune responses to O-specific polysaccharide (OSP) in North American adults infected with Vibrio cholerae O1 Inaba
Source: PLoS Negl Trop Dis. 2019 Nov 19;13(11):e0007874. doi: 10.1371/journal.pntd.0007874 (PMC6863522; doi:10.1371/journal.pntd.0007874)
Supplement: S1 Table — (DOCX) [file pntd.0007874.s001.docx]

| Time point in the North American volunteers (n) | GMT values with 95% confidence interval of vibriocidal titer | Time point in the Bangladeshi patients (n) | GMT values with 95% confidence interval of vibriocidal titer |
| --- | --- | --- | --- |
| Day 0 (38) | 22 (15-34) | Day 2 (38) | 29 (19-44) |
| Day 10 (38) | 7241 (5593-9375) | Day 7 (38) | 1810 (1178-2782) |
| Day 28 (38) | 3964 (3018-5207) | Day 21/30 (37) | 664 (430-1027) |
| Day 90 (16) | 562 (282-1121) | Day 90 (13) | 58 (29-117) |
| Day 170 (22) | 482 (331-702) | Day 180 (13) | 42 (28-63) |
