# Supplementary material for: Immune responses to O-specific polysaccharide (OSP) in North American adults infected with Vibrio cholerae O1 Inaba
Source: PLoS Negl Trop Dis. 2019 Nov 19;13(11):e0007874. doi: 10.1371/journal.pntd.0007874 (PMC6863522; doi:10.1371/journal.pntd.0007874)
Supplement: S2 Table — (DOCX) [file pntd.0007874.s002.docx]

| Time point in the North American volunteers (n) | GMT values with 95% confidence interval of vibriocidal titer | Time point in the Bangladeshi patients (n) | GMT values with 95% confidence interval of vibriocidal titer |
| --- | --- | --- | --- |
| Day 0 (38) | 20 (14-28) | Day 2 (38) | 46 (29-74) |
| Day 10 (38) | 1402 (860-2287) | Day 7 (38) | 1402 (900-2186) |
| Day 28 (38) | 841 (514-1378) | Day 21/30 (37) | 628 (406-973) |
| Day 90 (16) | 113 (63-204) | Day 90 (13) | 80 (38-169) |
| Day 170 (22) | 141 (78-254) | Day 180 (13) | 65 (36-115) |
