# Supplementary figures and images for: Immune responses to O-specific polysaccharide (OSP) in North American adults infected with Vibrio cholerae O1 Inaba
Source: PLoS Negl Trop Dis. 2019 Nov 19;13(11):e0007874. doi: 10.1371/journal.pntd.0007874 (PMC6863522; doi:10.1371/journal.pntd.0007874)

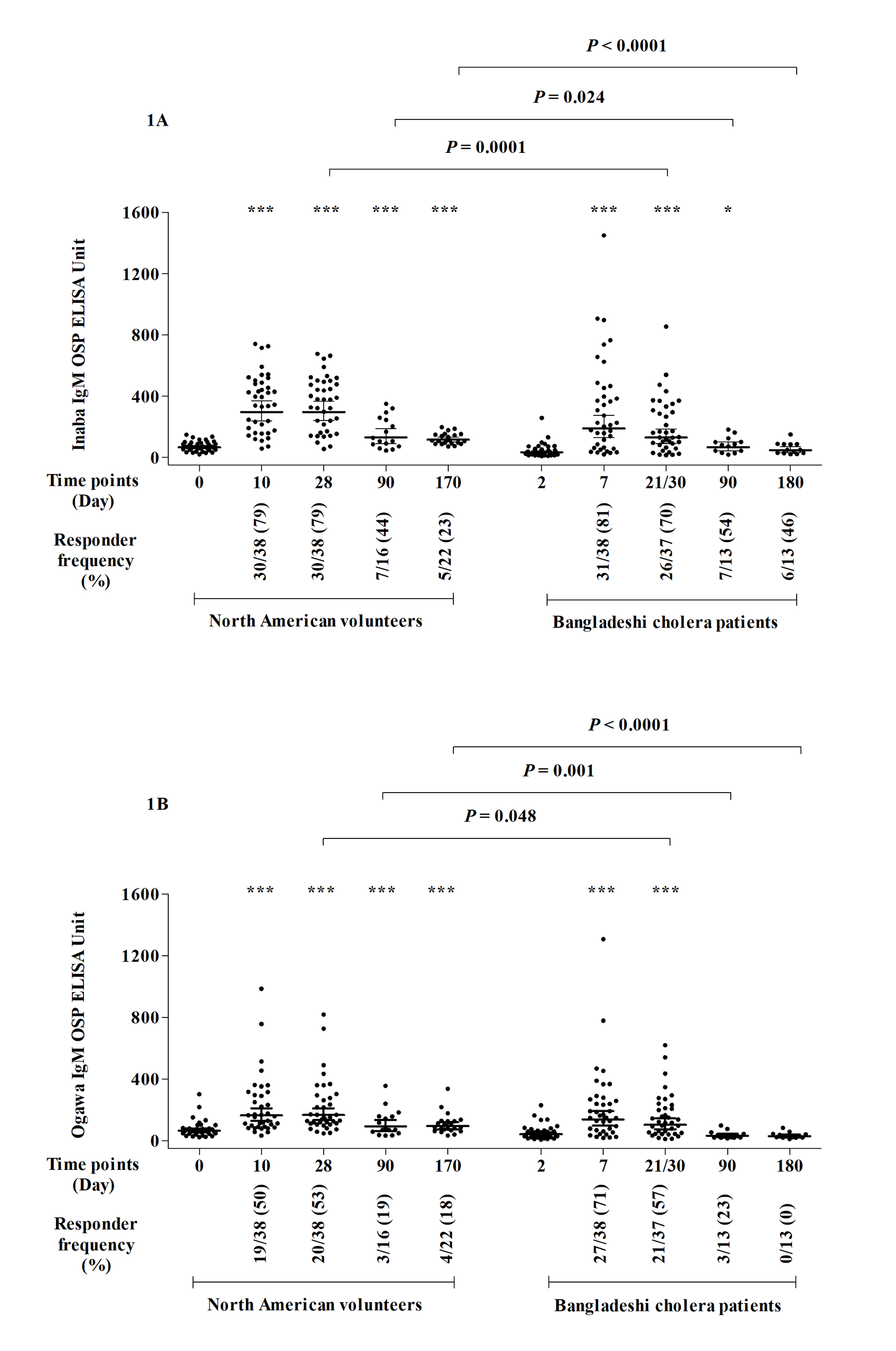

Supplement: S1 Fig — In the two cohorts, the numbers of patients with sample measurements are shown below the x axes. (1A) Inaba OSP-specific IgM antibody responses and (1B) Ogawa OSP-specific IgM antibody responses. X axis indicates the time points of samples, and the Y-axis denotes IgM antibody responses. Each single dot indicates an individual antibody response, horizontal bars indicate the geometric mean (GM), and error bars indicate 95% confidence intervals. P values represent statistical differences of the mean between the country groups. Asterisks represent statistically significant differences of immune responses within the country group compared to baseline (*** P ≤ 0.001, ** P ≤ 0.01, * P ≤ 0.05.). Responder frequencies (defined in text) are shown in parentheses below the X-axes. (TIF) [file pntd.0007874.s003.tif]

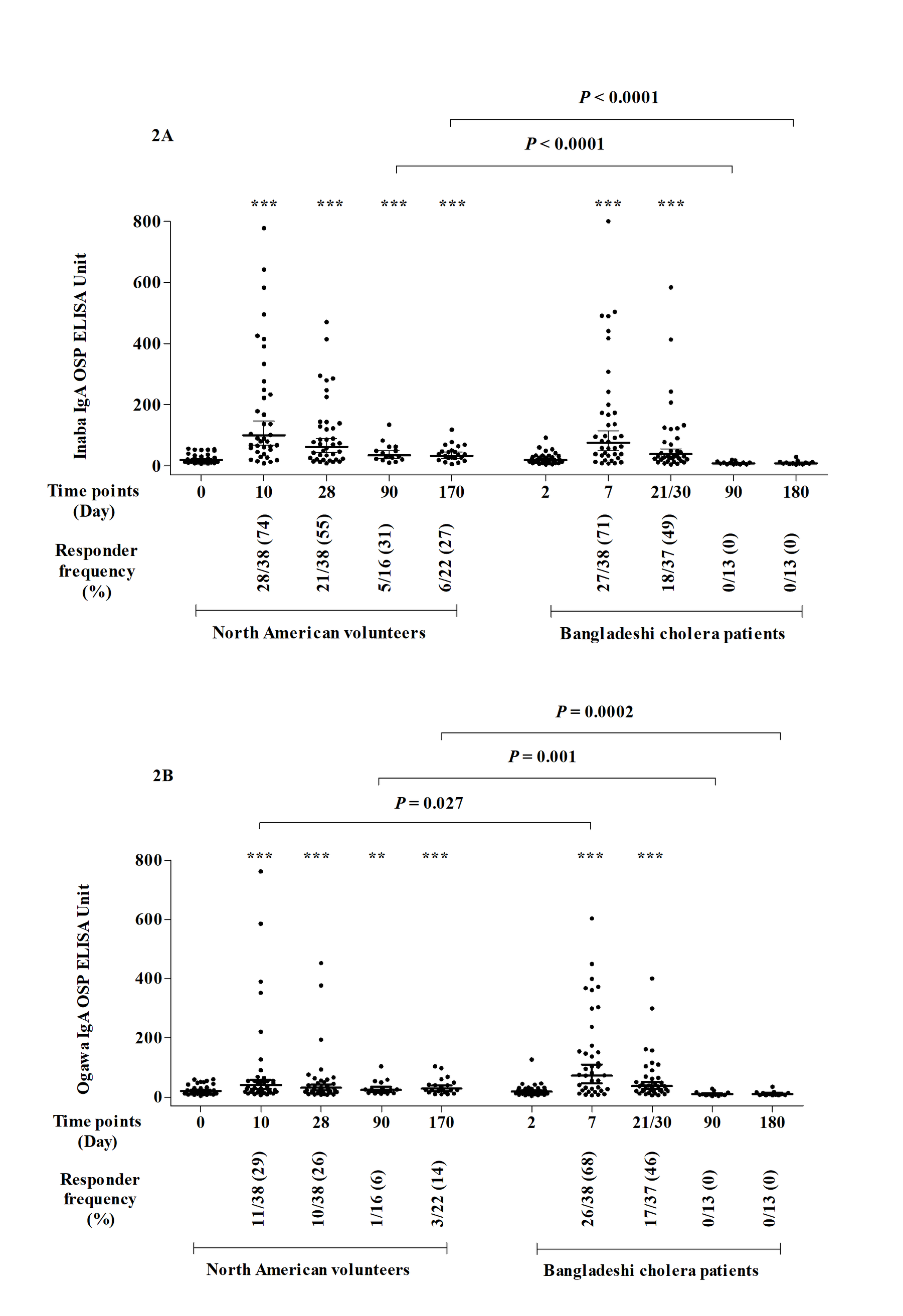

Supplement: S2 Fig — In the two cohorts, the numbers of patients with sample measurements are shown below the x axes. (2A) Inaba OSP-specific IgA antibody responses and (2B) Ogawa OSP-specific IgA antibody responses. X axes indicate the time points of samples, while the Y-axes denote IgA antibody responses. Each single dot indicates an individual antibody response, horizontal bars indicate the geometric mean (GM), and error bars indicate 95% confidence intervals. P values represent statistical differences of the mean between the country groups. Asterisks represent statistically significant differences of immune responses compared to baseline within the country group (*** P ≤ 0.001, ** P ≤ 0.01, * P ≤ 0.05). Responder frequencies (defined in text) are shown in parentheses below the X-axes. (TIF) [file pntd.0007874.s004.tif]

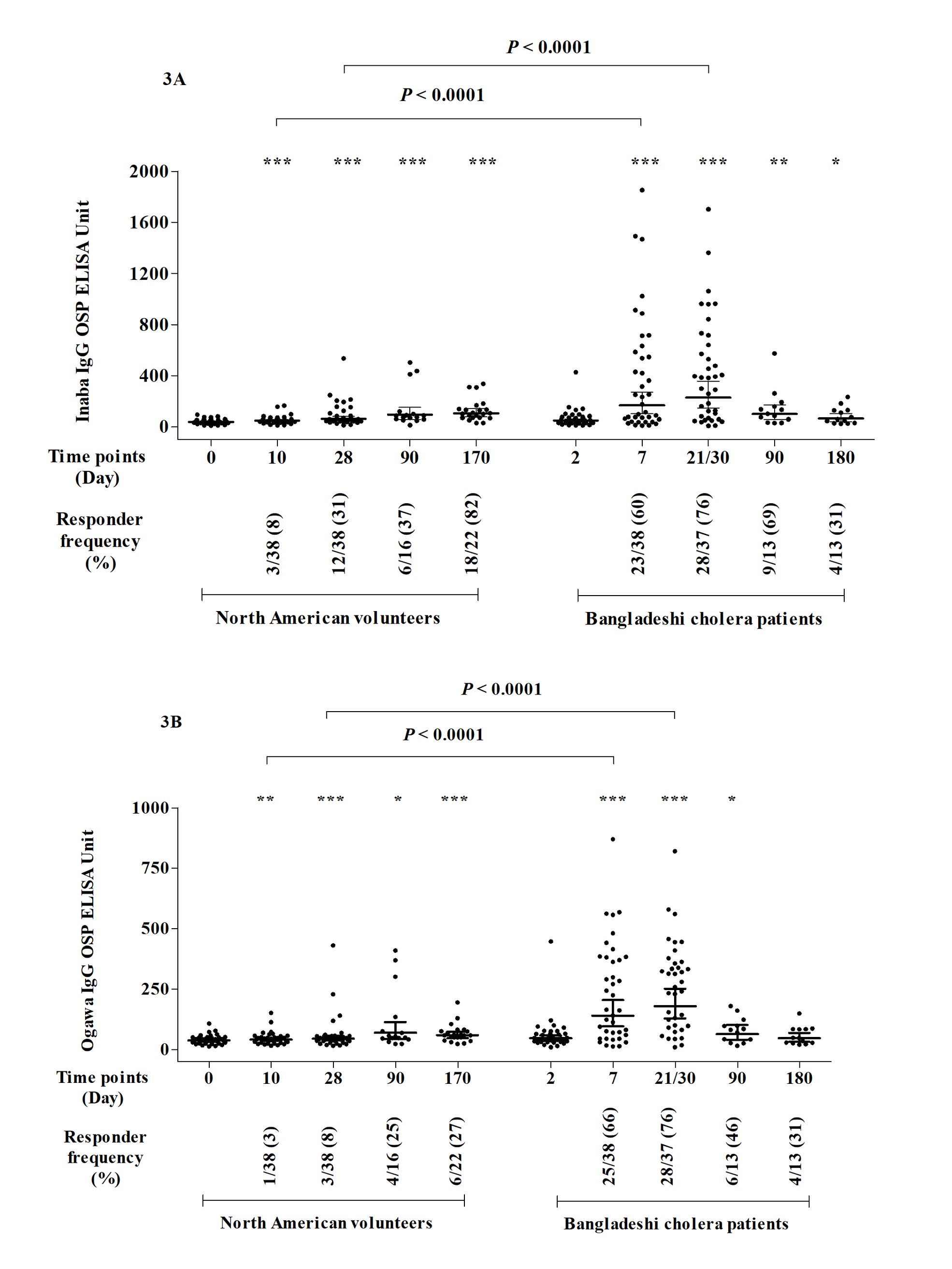

Supplement: S3 Fig — In the two cohorts, the numbers of patients with sample measurements are shown below the x axes. (3A) Inaba OSP-specific IgG antibody responses and (3B) Ogawa OSP-specific IgG antibody responses. X axes indicate the time points of samples, while the Y-axes denote IgG antibody responses. Each single dot indicates an individual antibody response, horizontal bars indicate the geometric mean (GM), and error bars indicate 95% confidence intervals. P values represent statistical differences of the mean between the country groups. Asterisks represent statistically significant differences of immune responses compared to baseline within country group (*** P ≤ 0.001, ** P ≤ 0.01, * P ≤ 0.05). Responder frequencies (defined in text) are shown in parentheses below the X-axes. (TIF) [file pntd.0007874.s005.tif]

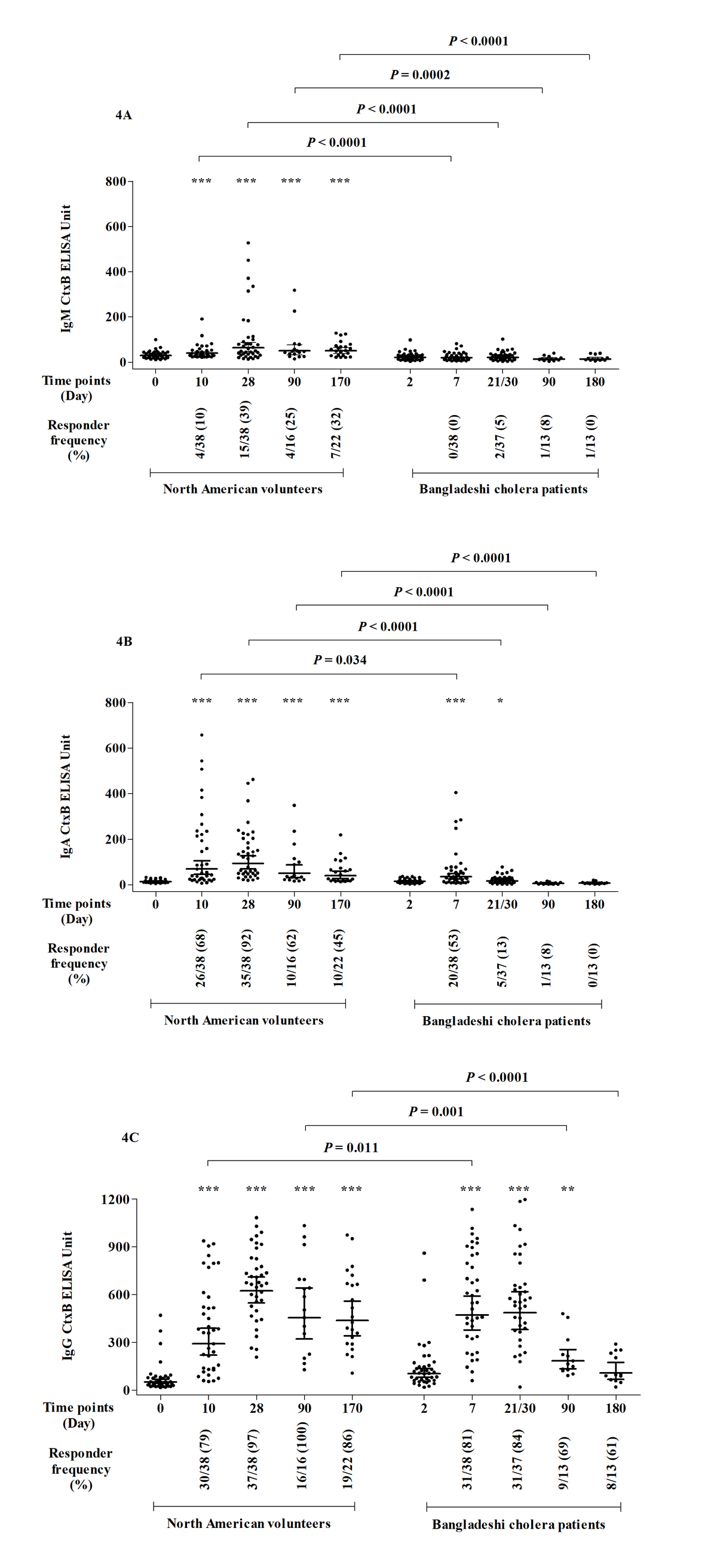

Supplement: S4 Fig — In the two cohorts, the numbers of patients with sample measurements are shown below the x axes. (4A) CtxB-specific IgM antibody responses, (4B) CtxB-specific IgA antibody responses and (4C) CtxB-specific IgG antibody responses. X axes indicate the time points of samples, while the Y-axes denote antibody responses. Each single dot indicates an individual antibody response, horizontal bars indicate the geometric mean (GM), and error bars indicate 95% confidence intervals. P values represent statistical differences of the mean between the country groups. Asterisks represent statistically significant differences of immune responses compared to baseline within country group (*** P ≤ 0.001, ** P ≤ 0.01, * P ≤ 0.05). Responder frequencies (defined in text) are shown in parentheses below the X-axes. (TIF) [file pntd.0007874.s006.tif]

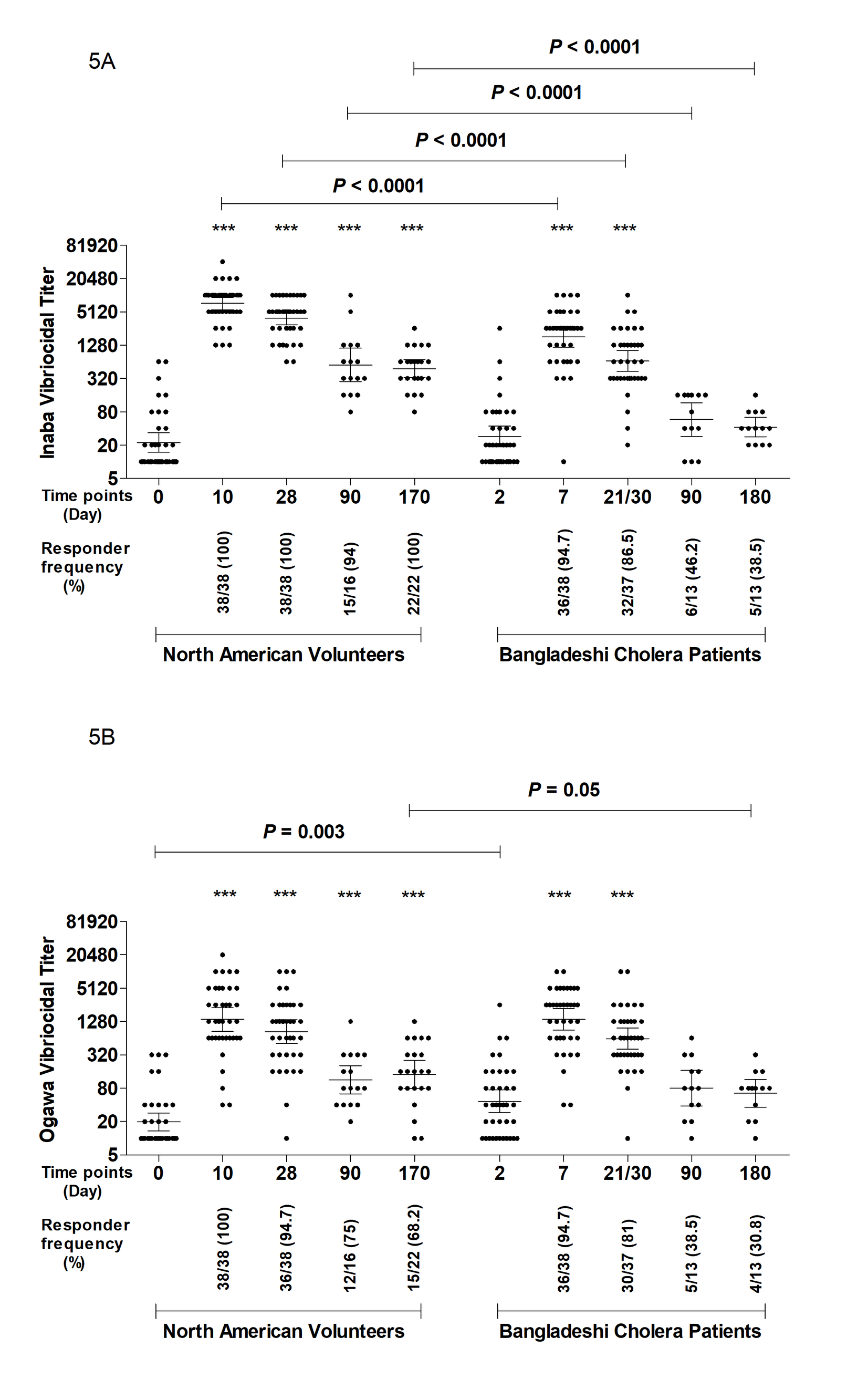

Supplement: S5 Fig — The time points of samples are plotted on the X axis while vibriocidal antibody responses are plotted on the Y-axis. (5A) Inaba vibriocidal antibody responses and (5B) Ogawa vibriocidal antibody responses. Each single dot indicates an individual vibriocidal antibody reciprocal end-dilution titer. Horizontal bars indicate the Geometric Mean (GM) and error bars indicate 95% Confidence Intervals (CI). P values represent statistical differences of the mean vibriocidal responses between the two country groups. Asterisks represent statistically significant differences of vibriocidal responses compared to the baseline time point within a country group (*** P ≤ 0.001, * P ≤ 0.05). Responder frequencies (defined in text) are also presented in parentheses below the X-axes. (TIF) [file pntd.0007874.s007.tif]

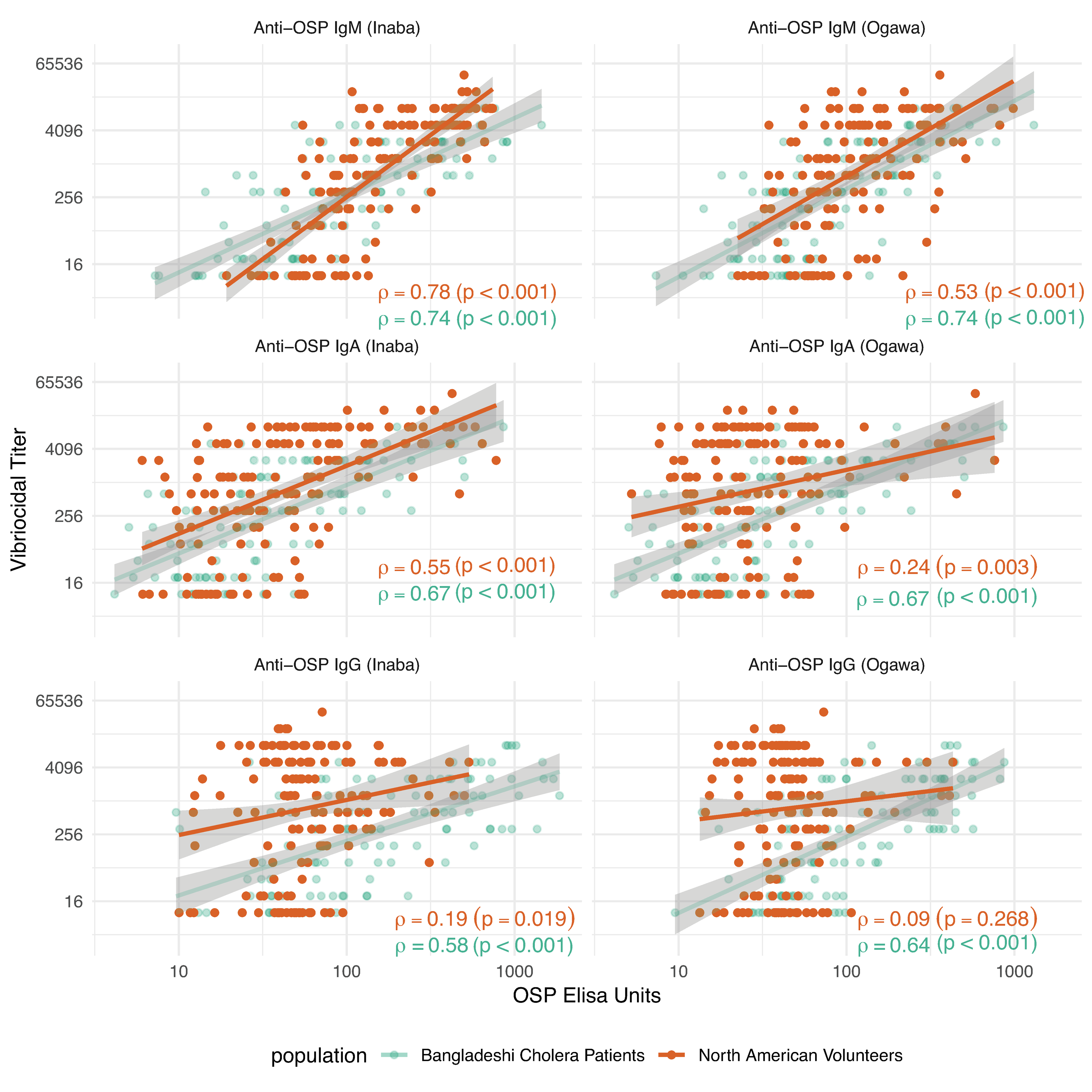

Supplement: S6 Fig — Lines represent simple linear regression lines with asymptotic 95% confidence intervals shown as grey envelopes. Pearson’s correlation coefficient and corresponding p-values are shown in each panel. Left panels compare Inaba OSP-specific IgM, IgA, and IgG to Inaba vibriocidal responses; Right panels compare Ogawa OSP-specific IgM, IgA, and IgG to Ogawa vibriocidal responses. (TIF) [file pntd.0007874.s008.tif]

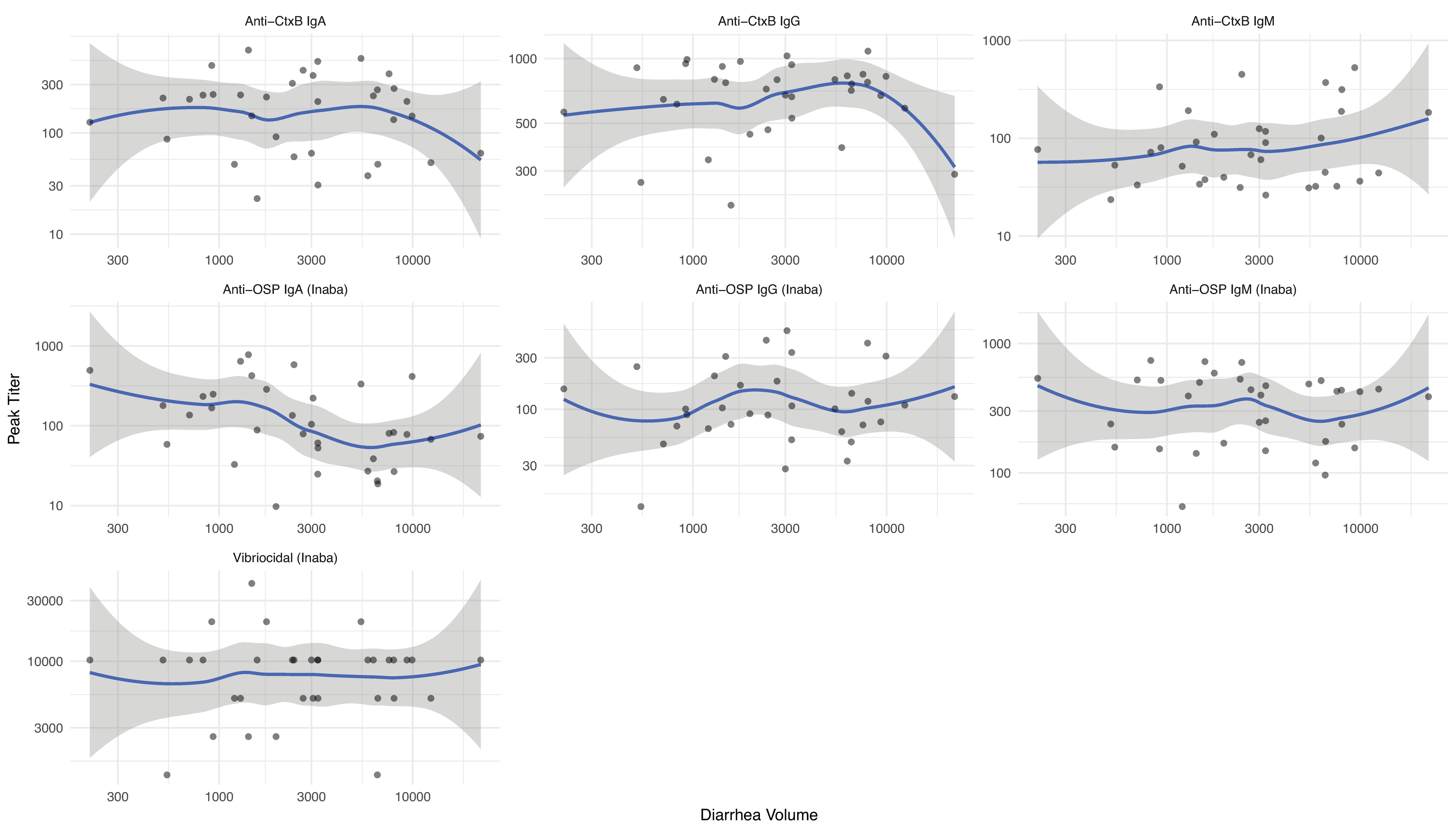

Supplement: S7 Fig — Blue lines show smoothed LOESS estimates and 95% confidence intervals. (TIF) [file pntd.0007874.s009.tif]

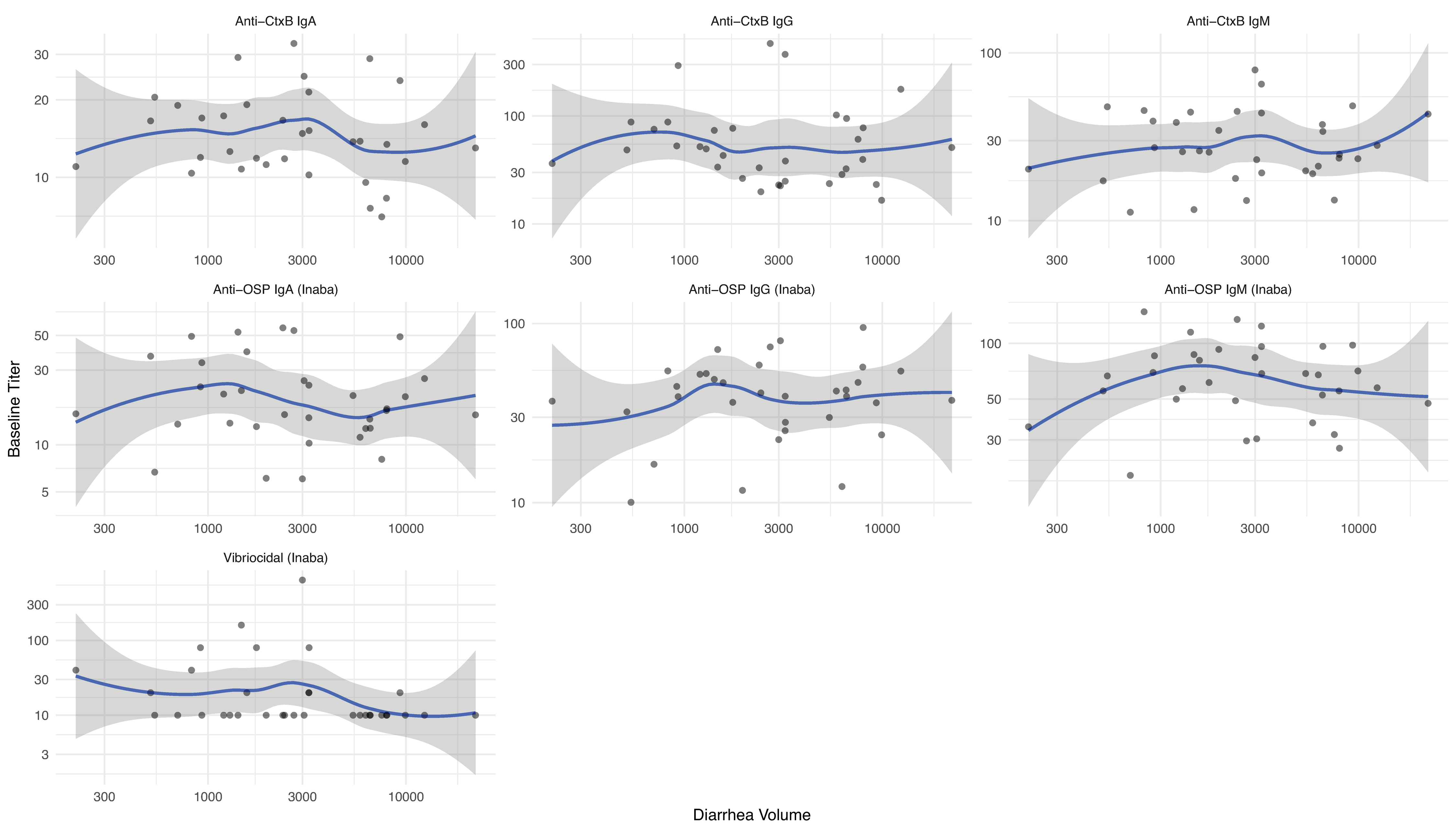

Supplement: S8 Fig — Blue lines show smoothed LOESS estimates and 95% confidence intervals. (TIF) [file pntd.0007874.s010.tif]

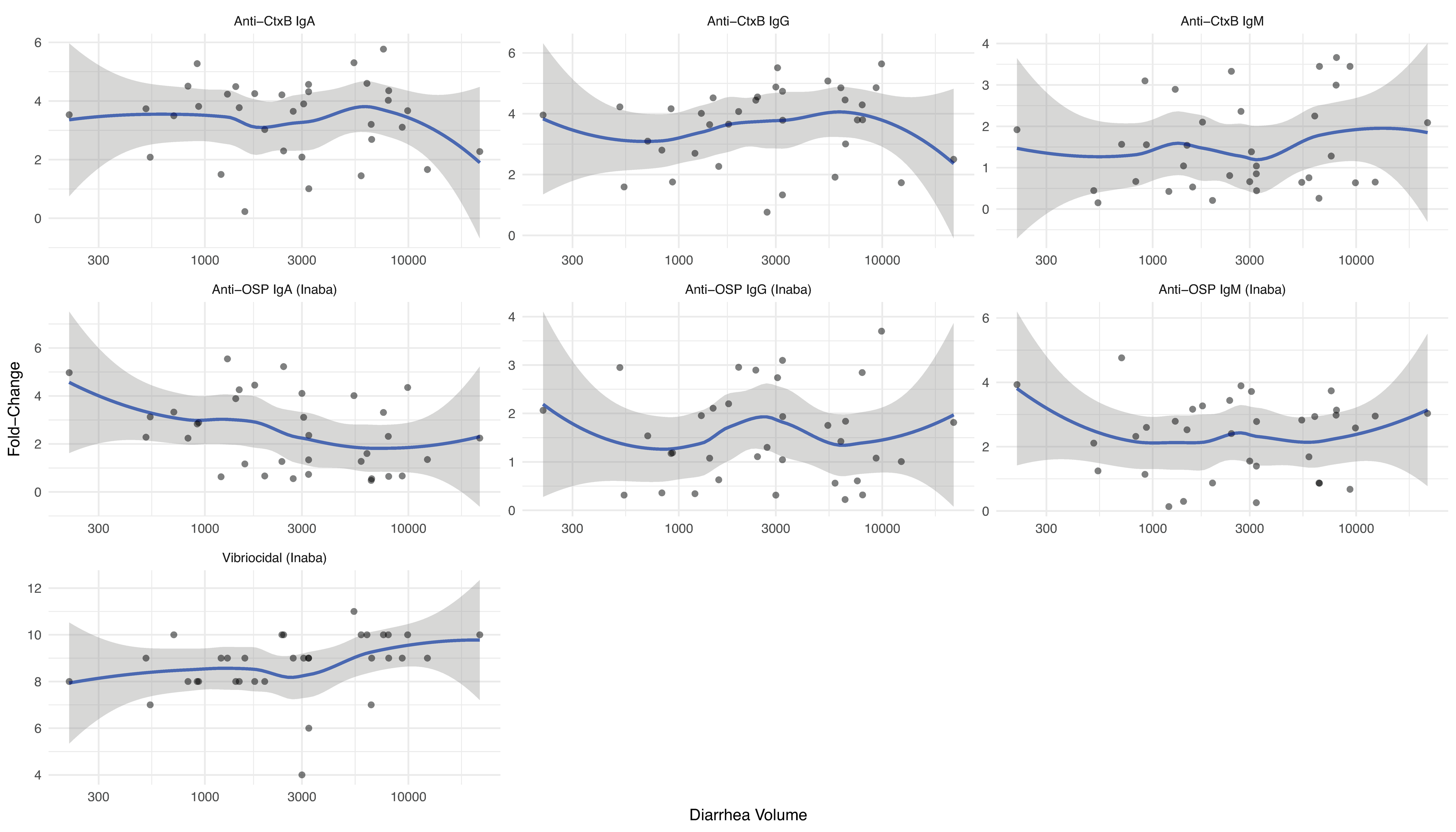

Supplement: S9 Fig — Blue lines show smoothed LOESS estimates and 95% confidence intervals. (TIF) [file pntd.0007874.s011.tif]

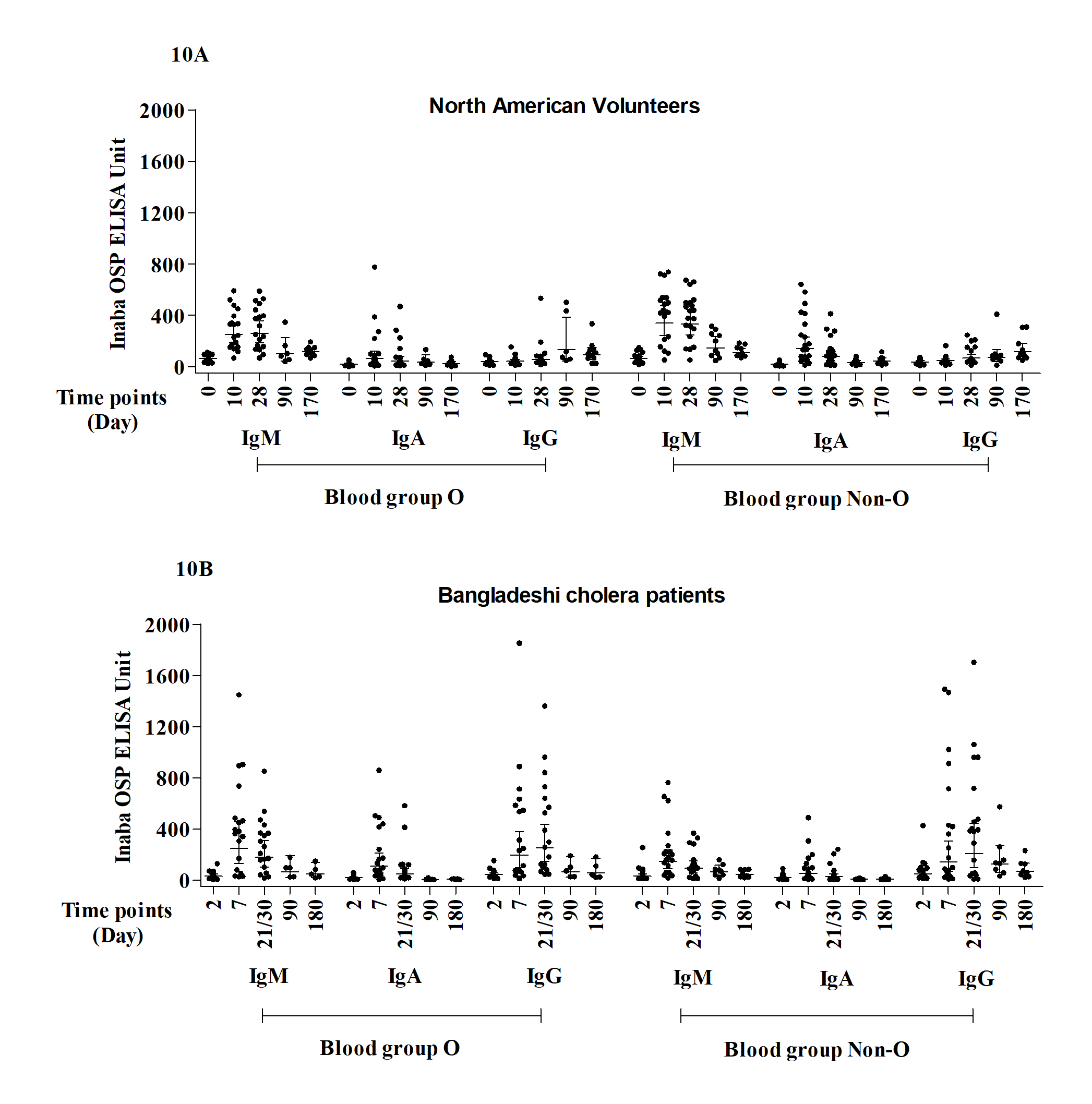

Supplement: S10 Fig — X axis indicates the time points of samples, while Y-axis denotes OSP-specific antibody responses. Each single dot indicates an individual OSP antibody value, horizontal bars indicate the geometric mean (GM), and error bars indicate 95% confidence intervals. (TIF) [file pntd.0007874.s012.tif]

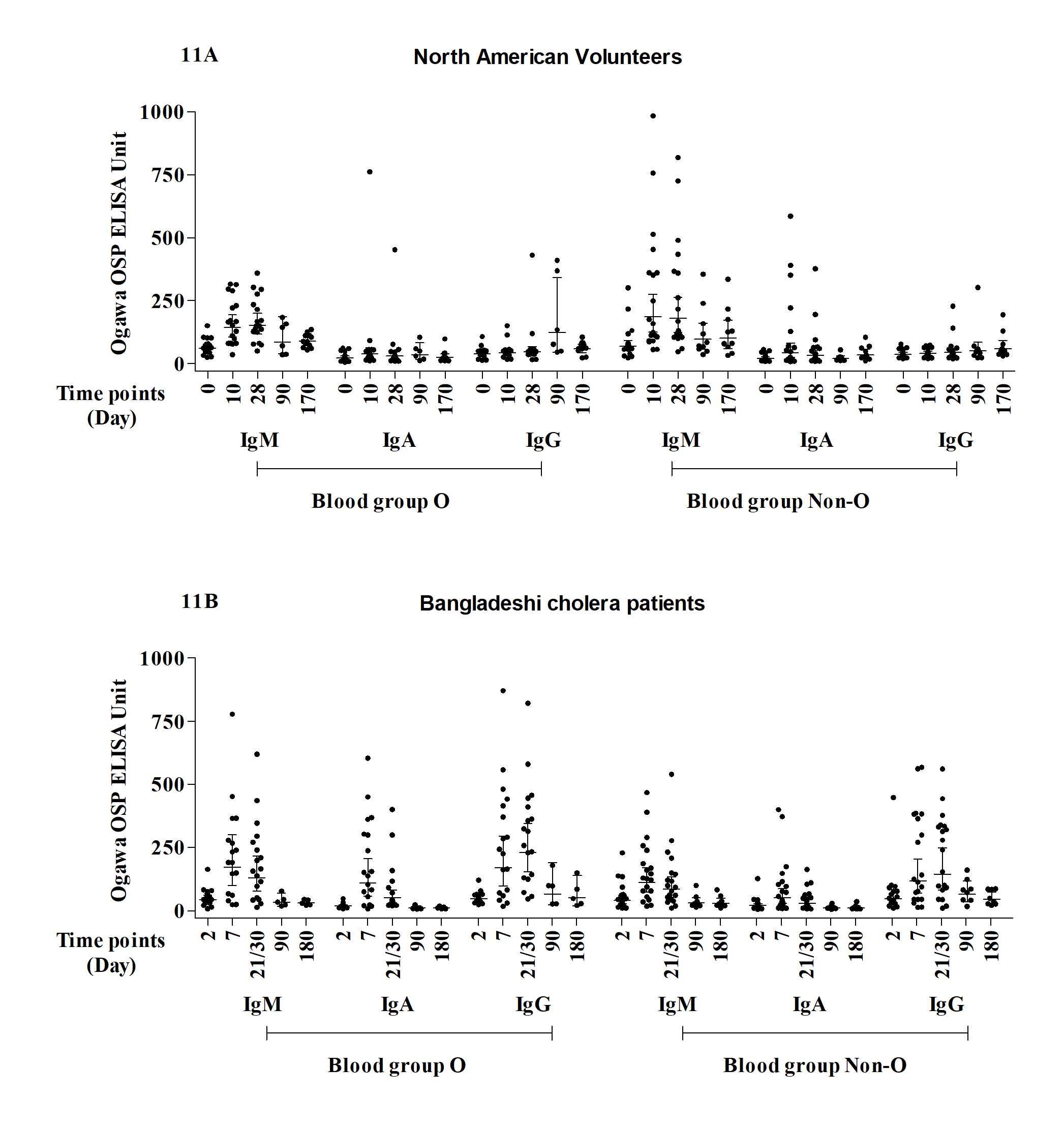

Supplement: S11 Fig — X axis indicates the time points of samples, while Y-axis denotes OSP-specific antibody responses. Each single dot indicates an individual OSP antibody value, horizontal bars indicate the geometric mean (GM), and error bars indicate 95% confidence intervals. (TIF) [file pntd.0007874.s013.tif]

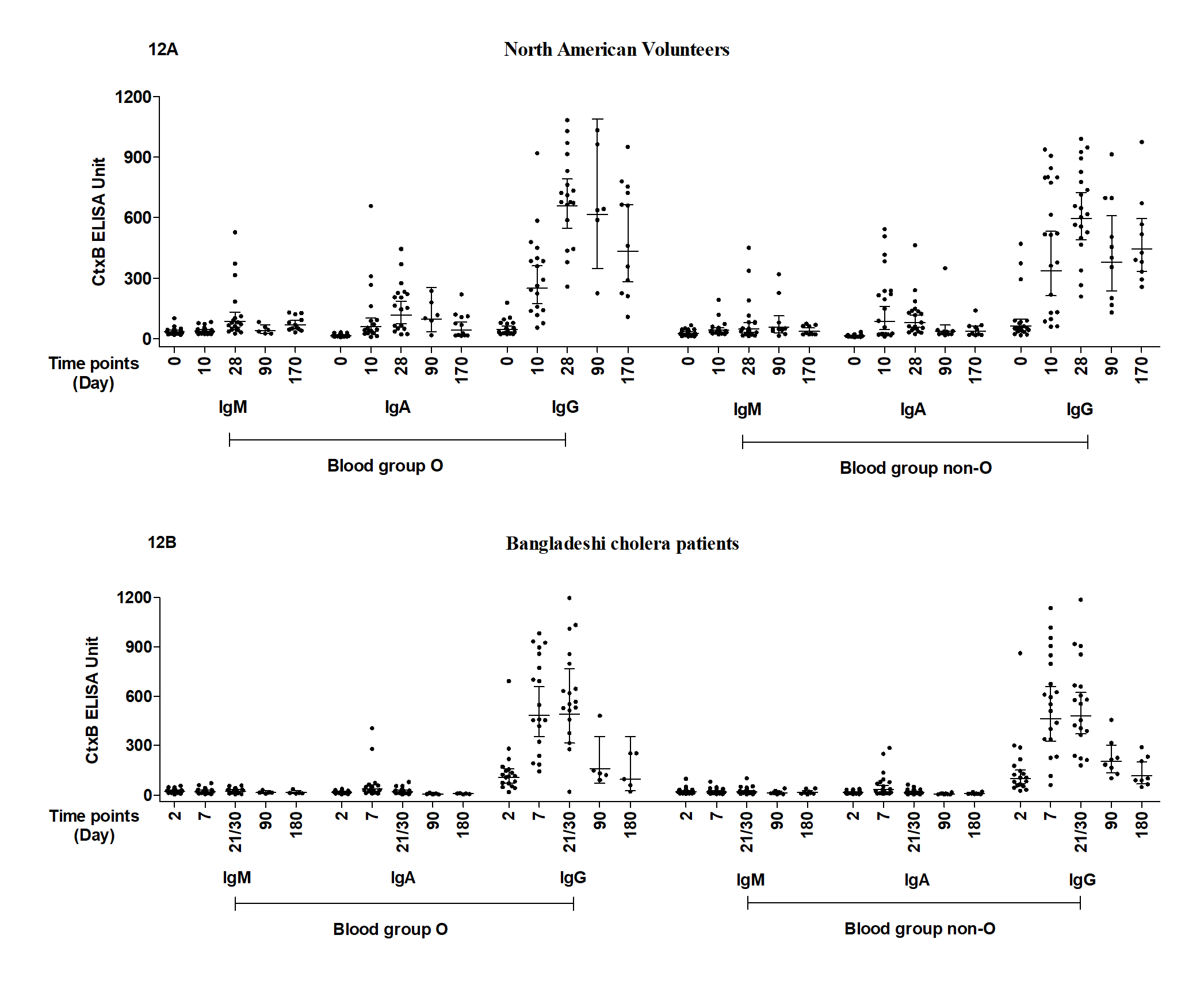

Supplement: S12 Fig — X axis indicates the time points of samples, while Y-axis denotes CtxB-specific antibody responses. Each single dot indicates an individual CtxB antibody value, horizontal bars indicate the geometric mean (GM), and error bars indicate 95% confidence intervals. (TIF) [file pntd.0007874.s014.tif]

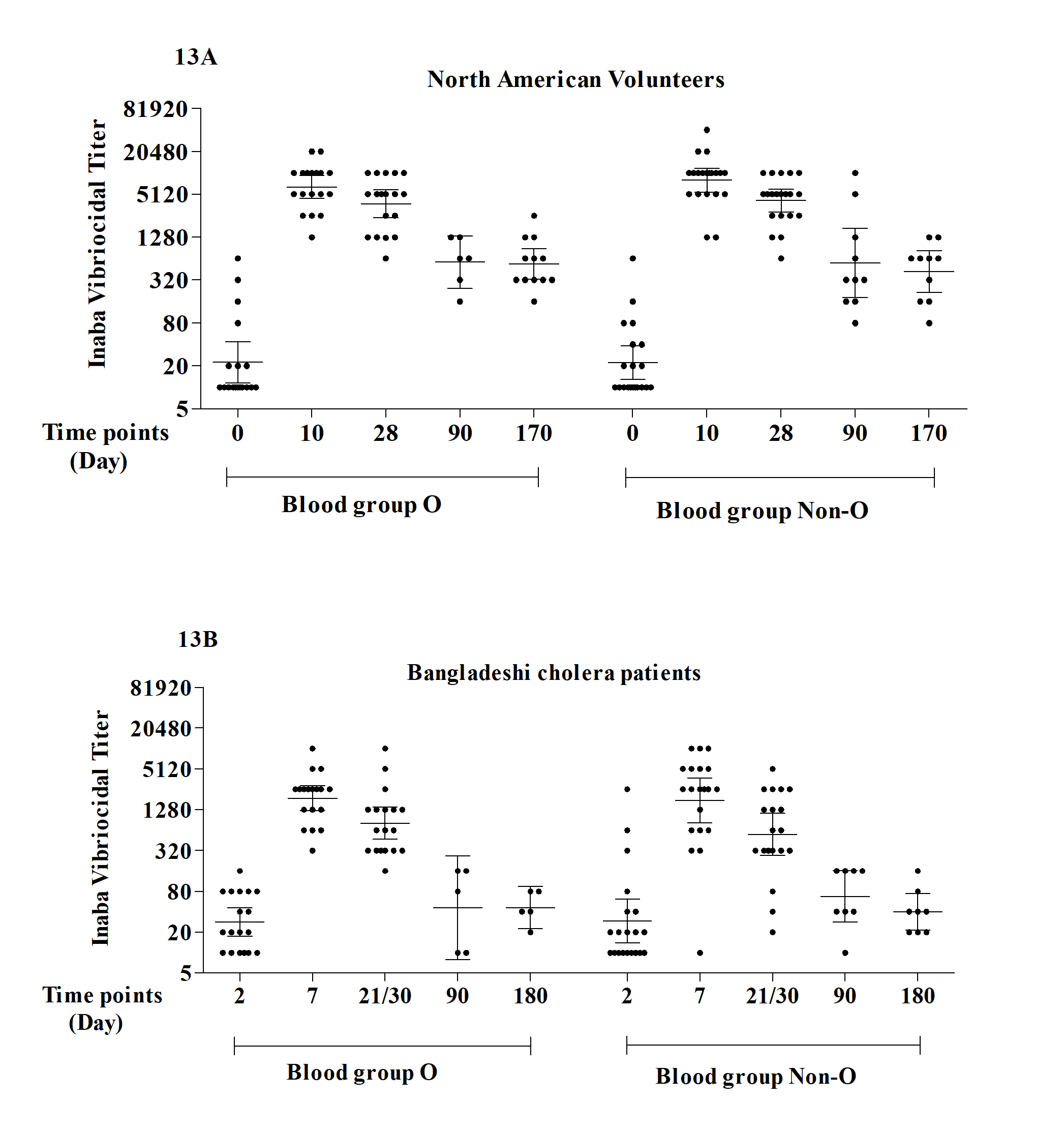

Supplement: S13 Fig — X axis indicates the time points of samples, while Y-axis denotes Inaba vibriocidal antibody responses. Each single dot indicates an individual vibriocidal antibody titer, horizontal bars indicate the geometric mean (GM), and error bars indicate 95% confidence intervals. (TIF) [file pntd.0007874.s015.tif]

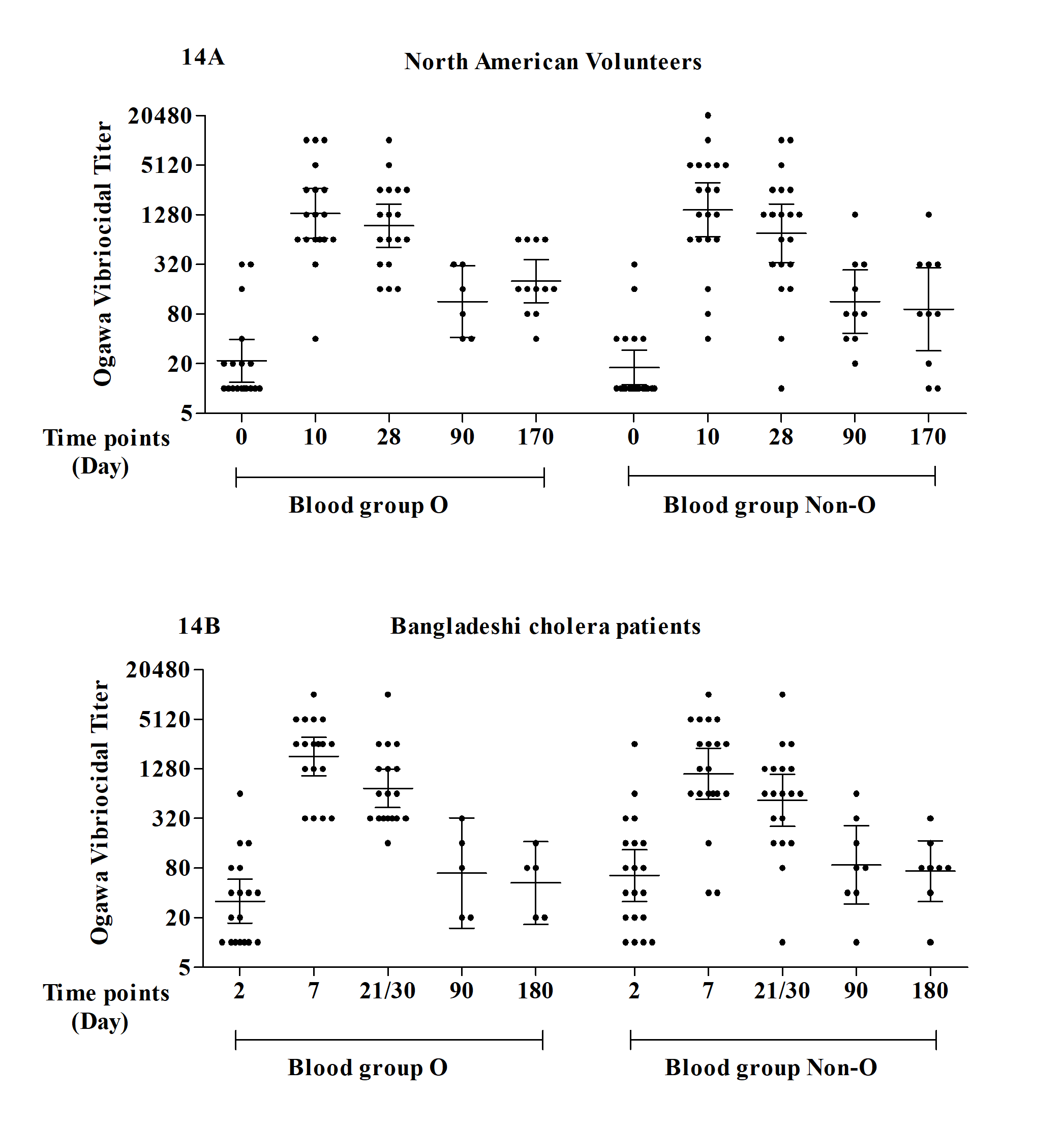

Supplement: S14 Fig — X axis indicates the time points of samples, while Y-axis denotes Ogawa vibriocidal antibody responses. Each single dot indicates an individual vibriocidal antibody titer, horizontal bars indicate the geometric mean (GM), and error bars indicate 95% confidence intervals. (TIF) [file pntd.0007874.s016.tif]
